# Protein phosphorylation in neurodegeneration: friend or foe?

**DOI:** 10.3389/fnmol.2014.00042

**Published:** 2014-05-13

**Authors:** Sandra Tenreiro, Katrin Eckermann, Tiago F. Outeiro

**Affiliations:** ^1^Cell and Molecular Neuroscience Unit, Instituto de Medicina MolecularLisboa, Portugal; ^2^Department of Neurology, Center for Nanoscale Microscopy and Molecular Physiology of the Brain, University Medical Center GöttingenGöttingen, Germany; ^3^Instituto de Fisiologia, Faculdade de Medicina da Universidade de LisboaLisboa, Portugal; ^4^Department of NeuroDegeneration and Restorative Research, Center for Nanoscale Microscopy and Molecular Physiology of the Brain, University Medical Center GöttingenGöttingen, Germany

**Keywords:** alpha-synuclein, tau, Parkinson's disease, Alzheimer's disease, phosphorylation, neurodegeneration

## Abstract

Protein misfolding and aggregation is a common hallmark in neurodegenerative disorders, including Alzheimer's disease (AD), Parkinson's disease (PD), and fronto-temporal dementia (FTD). In these disorders, the misfolding and aggregation of specific proteins occurs alongside neuronal degeneration in somewhat specific brain areas, depending on the disorder and the stage of the disease. However, we still do not fully understand the mechanisms governing protein aggregation, and whether this constitutes a protective or detrimental process. In PD, alpha-synuclein (aSyn) forms protein aggregates, known as Lewy bodies, and is phosphorylated at serine 129. Other residues have also been shown to be phosphorylated, but the significance of phosphorylation in the biology and pathophysiology of the protein is still controversial. In AD and in FTD, hyperphosphorylation of tau protein causes its misfolding and aggregation. Again, our understanding of the precise consequences of tau phosphorylation in the biology and pathophysiology of the protein is still limited. Through the use of a variety of model organisms and technical approaches, we are now gaining stronger insight into the effects of phosphorylation in the behavior of these proteins. In this review, we cover recent findings in the field and discuss how targeting phosphorylation events might be used for therapeutic intervention in these devastating diseases of the nervous system.

## Neurodegenerative disorders as protein aggregopathies

Neurodegenerative disorders, such as Parkinson's disease (PD), Alzheimer's disease (AD) and frontotemporal dementia (FTD), result from the progressive loss of specific neuronal populations leading to the progressive appearance of the clinical symptoms that are characteristic of each disorder. Current treatments for these disorders are palliative rather than curative and their effectiveness is still far from satisfactory. Thus, tremendous efforts are underway to elucidate the causes underlying these disorders and to find a cure. From a molecular perspective, the common hallmark of neurodegenerative disorders is the misfolding and aberrant aggregation of proteins in amyloid-like beta-sheet filaments. This feature is not only characteristic of classic neurodegenerative disorders but also of prion disorders and other amyloidosis inside and outside the central nervous system, suggesting that neurodegenerative disorders are part of a much greater superfamily of protein misfolding disorders, or aggregopathies (Frost and Diamond, 2010).

While it is clear that protein misfolding and aggregation are pathological hallmarks of neurodegenerative disorders, the precise mechanisms linking protein aggregation and neurotoxicity are largely unknown. Protein aggregates are dynamic structures, allowing small soluble species to detach or attach from or to larger protein inclusions relatively easily (Kim et al., 2002). As a result of this dynamism, protein inclusions have variable solubility, stability and size. Big, insoluble protein inclusions inside or outside neurons were initially thought to be neurotoxic. However, current evidence indicates that they might be rather neuroprotective (Arrasate et al., 2004; Bodner et al., 2006), and that the smaller, more soluble oligomers are the ones that exert neurotoxicity. A consensus in this matter remains to be reached but, regardless of the nature of the toxic and non-toxic species, unraveling the mechanisms determining protein aggregation is absolutely necessary for the understanding, diagnosis and treatment of neurodegenerative disorders.

## Posttranslational modifications as modulators of protein fate

**Figure 1:**
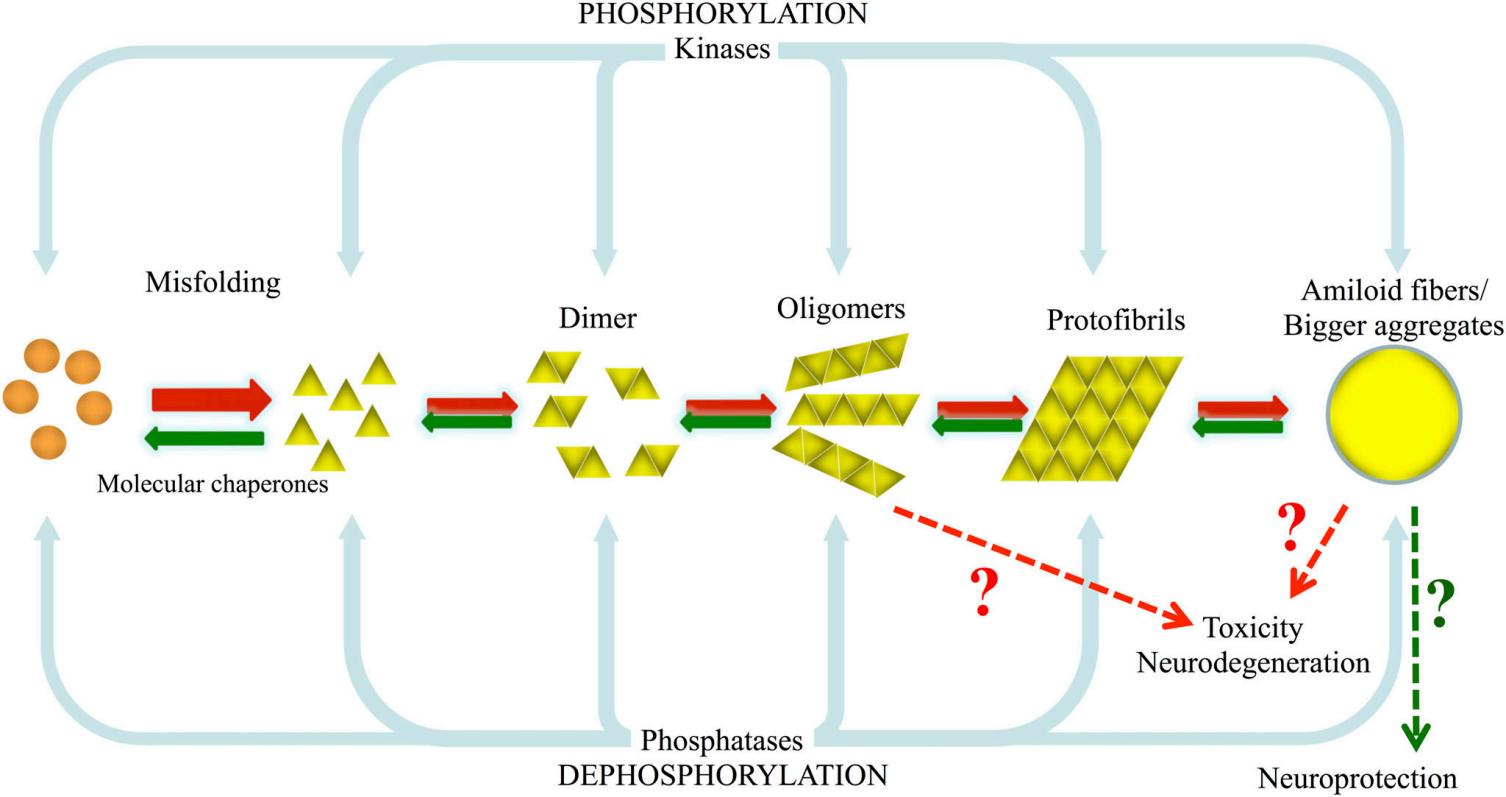
**Model of aSyn and tau misfolding and aggregation, and the involvement of kinases and phosphatases on their phosphorylation/dephosphorylation**. Under pathological conditions, due to genetic or environmental factors such as exposure to pesticides, normal highly soluble aSyn and tau misfold and are converted into pathological oligomers and larger species that fibrillize and deposit into inclusion bodies as LBs and Lewy neurites and into PHFs and NFTs. In this situation the normal cellular quality-control systems (molecular chaperones, ubiquitin proteasome system (UPS), phagosome/lysosome system) are not able to counteract and prevent or reverse protein misfolding or eliminate proteins that have misfolded or assembled into pathological aggregates and amyloid fibrils.

Protein aggregation can be regulated by various cellular events including different types of stress, molecular crowding, or the local micro-environment. In addition, diverse posttranslational modifications (PTMs), such as phosphorylation, ubiquitination or sumoylation, which alter the conformation and/or biological function of proteins, can also affect protein folding and aggregation, and thereby play a critical role in neurodegenerative disorders. For example, ubiquitination can direct proteins for either degradation by the proteasome or to certain subcellular compartments, and glycosylation is related to the secretion of proteins to the extracellular medium. Both processes could therefore influence protein concentration, folding, localization and, ultimately, aggregation. Phosphorylation can also affect protein conformation, function and fate in many different ways: it may be required for proper protein folding; it may induce conformational changes that can result in lower or higher catalytic activity; it may precede or function as a recognition signal for further modifications, such as ubiquitination; it may alter the subcellular localization of the protein; and it may modify protein-protein interactions (Salazar and Hofer, 2009). In the particular case of neurodegenerative and protein misfolding diseases, phosphorylation has been shown to be involved in both protein aggregation and toxicity, as illustrated by the paradigmatic examples described below (Figure 1).

## Phosphorylation in parkinson's disease and other synucleinopathies

Alpha-synuclein (aSyn) is the main protein component of Lewy bodies (LBs), the typical pathological hallmarks of PD and other disorders collectively known as synucleinopathies. The vast majority of PD cases are sporadic, multiplications and missense mutations in the gene encoding for aSyn have been associated with familial forms of PD (Polymeropoulos et al., 1997; Kruger et al., 1998; Singleton et al., 2003; Chartier-Harlin et al., 2004; Zarranz et al., 2004; Appel-Cresswell et al., 2013; Kiely et al., 2013; Lesage et al., 2013).

Several PTMs have already been identified in aSyn. These include ubiquitination (Shimura et al., 2001), phosphorylation at S129 (Fujiwara et al., 2002), a C-terminal truncation (Li et al., 2005), nitration on tyrosine residues (Giasson et al., 2000), glycosylation, and SUMO modification (Dorval and Fraser, 2006). However, most attention was devoted to phosphorylation at S129 (pS129). While only 4% of the soluble, monomeric aSyn appears phosphorylated under physiological conditions *in vivo*, approximately 90% is phosphorylated in LB lesions (Fujiwara et al., 2002; Anderson et al., 2006), suggesting a close relationship between aSyn phosphorylation at S129 and its aggregation. In particular, pS129 aSyn is found in LBs occurring and other pathogenic inclusions found in substantia nigra of PD patients (Fujiwara et al., 2002; Saito et al., 2003; Anderson et al., 2006) as well as in different brain regions of patients suffering from other synucleinopathies, such as dementia with LBs (DLB), multiple system atrophy (Fujiwara et al., 2002; Kahle et al., 2002; Saito et al., 2003; Nishie et al., 2004; Waxman and Giasson, 2008), Hallervorden-Spatz disease (Fujiwara et al., 2002), pure autonomic failure (Arai et al., 2000b), and LB variant of AD (LBVAD) (Waxman and Giasson, 2008). In addition, aSyn was found to be phosphorylated on S129 in transgenic mice expressing human mutant A30P, A53T, or WT aSyn (Kahle et al., 2002; Freichel et al., 2007; Wakamatsu et al., 2007).

**Figure 2:**
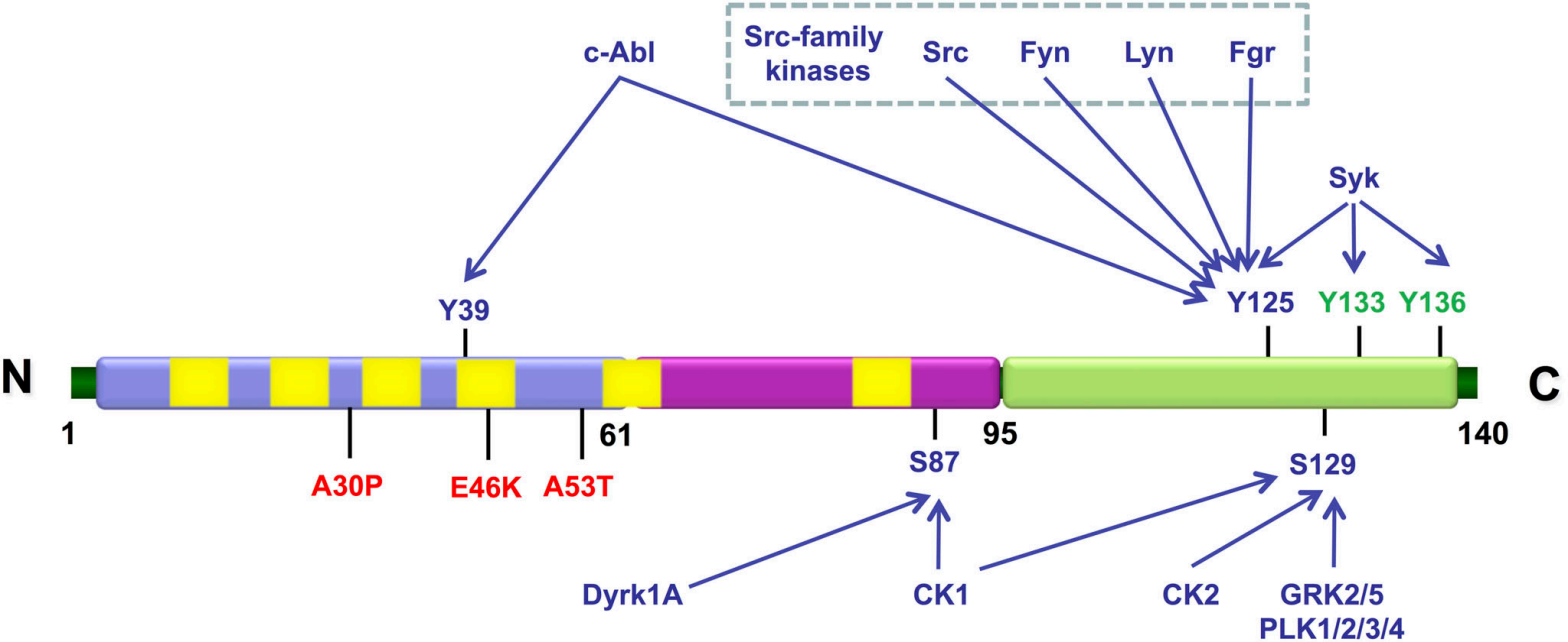
**Schematic representation illustrating the various residues in aSyn that can be phosphorylated *in vivo* (represented in blue) and *in vitro* (represented in green)**. The mutations associated with familial PD are shown in red. The N-terminal amphipathic region of the protein is represented in blue, the hydrophobic central region that contains the non-amyloid-β component (NAC) domain is represented in purple and the highly acidic C-terminal is represented in green. The imperfect KTKEGV repeats are represented in yellow. The kinases described as being able to phosphorylate each of the indicated residues are also indicated.

In addition to S129, other three serine, four tyrosine and ten threonine residues are putative sites of phosphorylation (Figure 2). These residues are mostly localized in the C-terminal region of the protein, with the exception of Y39 and S87. Increased levels of phosphorylated S87 (pS87) were also reported in synucleinopathies (Paleologou et al., 2010). Phosphorylation aSyn on tyrosine 39 (pY39) and 125 (pY125) was also reported in human brains but no correlation was established between increased levels of phosphorylation in these residues and the pathological condition (Chen et al., 2009; Mahul-Mellier et al., 2014). Other residues were found to be phosphorylated *in vitro* but it is unknown if their phosphorylation also occurs *in vivo*, even if in small extension.

### The role of phosphorylation on aSyn cytotoxicity and aggregation

The phosphorylation status of aSyn clearly influences its aggregation and toxicity, but it is still unclear whether phosphorylation promotes or prevents aggregation and toxicity. To better understand this trinomial relation, would also be important to clearly establish what are the toxic forms of aggregated aSyn, although recent studies suggest that the soluble oligomeric/protofibrillar species may be more toxic than larger aggregated forms of aSyn (Spillantini et al., 1997; Conway et al., 2001; El-Agnaf et al., 2003; Outeiro et al., 2007; Diogenes et al., 2012).

*In vitro* and *in vivo* studies, correlating phosphorylation of aSyn in several residues to its aggregation and/or toxicity, resulted in conflicting results (Table 1). Several of these studies employed S129A and S129D/E mutants, to block and mimic phosphorylation, respectively. Other studies modulated the levels of phosphorylation of aSyn by either co-expressing specific kinases or phosphatases, or by using kinase inhibitors (Table 1). Moreover, these studies included three types of assays: (i) *in vitro* biochemical studies; (ii) single cell models (yeast and mammalian cells); and (iii) animal models of PD (mice or rat models). In these studies the relation between toxicity and aggregation was not always explored (Table 1).

**Table 1:** **aSyn phosphorylation sites and effects**.

**aSyn residue**	**Kinase**	**Model**	**Cytotoxicity**	**Aggregation**	**References**
Y39	c-Abl	*In vitro* biochemical assay/M17 neuroblastoma cell lines/ primary cultures of mouse cortical neurons / mThy1 aSyn transgenic mice / Rat model involving viral delivery	c-Abl inhibition increases aSyn degradation by proteasome and autophagy pathways	–	Mahul-Mellier et al., 2014
S87	CK1	*In vitro* biochemical assay/K293 and PC12 cells	–	–	Okochi et al., 2000
	Dyrk1A	*In vitro* biochemical assay/SH-SY5Y and H19-7 cells	Increased pS87 increases citotoxicity	Increased pS87 increases aSyn aggregation in cultured cells	Kim et al., 2006
	CK1	*In vitro* biochemical assay/SH-SY5Y cells/ transgenic mice M20 and M83	–	pS87 reduce recombinant aSyn fibril formation	Waxman and Giasson, 2008
	CK1	*In vitro* biochemical assay/ transgenic mouse models of PD/LBD and MSA	–	S87E or pS87 blocks aSyn fibrillization	Paleologou et al., 2010
	–	Rat model involving viral delivery of WT, S87A, and S87E aSyn	S87E protects against aSyn induced toxicity by reducing dystrophic fibers, and motor impairment	S87E inhibits aSyn aggregation	Oueslati et al., 2012
Y125	Fyn	*In vitro* biochemical assay/COS7 cells	–	–	Nakamura et al., 2001
	Src, Fyn	*In vitro* biochemical assay/HEK293T cells	–	–	Ellis et al., 2001
	Src-family kinases	COS7 cells	–	–	Nakamura et al., 2001
	Syk, Lyn, Fgr	*In vitro* biochemical assay/SH-N-BE and CHO cells	–	Syk-mediated aSyn phosphorylation decreases oligomerization	Negro et al., 2002
	kinase shark (Syk *Drosophila* homolog)	*Drosophila*	Increased pY125 is protective; Y125F is toxic	Increased pY125 decreases aSyn oligomerization while Y125F increases it	Chau et al., 2009
	Fyn	*In vitro* biochemical assay	–	*in vitro* pY125 fibrillate similarly to WT aSyn while Y125F or Y125E fibrillate significantly slower than WT aSyn	Schreurs et al., 2014
	c-Abl	*In vitro* biochemical assay/M17 neuroblastoma cell lines/ primary cultures of mouse cortical neurons / mThy1 aSyn transgenic mice / Rat model involving viral delivery	c-Abl inhibition increases aSyn degradation by proteasome and autophagy pathways	–	Mahul-Mellier et al., 2014
S129	CK1, CK2	*In vitro* biochemical assay/K293 cells	–	–	Okochi et al., 2000
	CK1, CK2, Grk2, Grk5	*In vitro* biochemical assay/COS-1 cells	–	–	Pronin et al., 2000
	CK2	*In vitro* biochemical assay	–	pS129 increases aSyn fibrillization *in vitro*	Fujiwara et al., 2002
	PLK2	*In vitro* biochemical assay/HEK293 cells/Mouse	–	–	Inglis et al., 2009
	CK1, CK2	*In vitro* biochemical assay	–	pSer129 inhibits rather than promotes aSyn fibrillization; S129A promotes aSyn aggregation	Paleologou et al., 2008
	PLK2	*In vitro* biochemical assay	–	*in vitro* pS129, S129A or S129D fibrillate similarly to WT aSyn	Schreurs et al., 2014
	Yck1 and Yck2 yeast CK1 kinases	*S. cerevisiae*	pS129 by plasma membrane CK1 kinases correlates with aSyn toxicity	pS129 by plasma membrane CK1 kinases correlates with aSyn inclusion formation	Zabrocki et al., 2008
	–	*Schizosaccharomyces pombe* and*S. cerevisiae*	Neither S129A nor S129D mutants altered WT aSyn toxicity	Both S129A and S129D increased endomembrane association in *S. pombe*, but only S129D decreased plasma membrane association in *S. cerevisiae*	Fischer et al., 2009
	CK1	*S. cerevisiae*	Yck1 CK1 yeast ortholog phosphorylate S129 aSyn and attenuate aSyn toxicity by an S129 phosphorylation-independent mechanism; S129A increases aSyn toxicity in a yeast genetic context-dependent manner	S129A increases aSyn inclusion formation a in a yeast genetic context-dependent manner;	Sancenon et al., 2012
	PLK1, PLK2, PLK3, PLK4	*S. cerevisiae*, mouse CAD cathecolaminergic cells and human H4 neuroglioma cells	PLK2 increased aSyn cytotoxicity in yeast but by an S129 phosphorylation-independent mechanism phosphorylation	PLK2 promotes aSyn inclusion formation in yeast and in mammalian cells by an S129 phosphorylation-independent mechanism	Basso et al., 2013
	–	*S. cerevisiae*	S129A increases aSyn toxicity	S129A aSyn forms more inclusions and oligomeric species with higher molecular weight than the WT form	Tenreiro et al., 2014
	GRK5	HEK293, SH-SY5Y cells and primary neurons from the cerebral cortex of fetal mice	–	Increased aggregation by co-expression with GRK5	Arawaka et al., 2006
	CK2	293T, PC-12 and NS20Y cells stably or transiently transfected with synphilin-1, aSyn and/or CKII	–	S129A mutation does not influence aSyn aggregation with synphilin-1	Lee et al., 2004a
	–	293T cells stably transfected with synphilin-1 co-expressing aSyn WT or S129A	S129A mutation does not influence aSyn toxicity	S129A mutation does not influence aSyn aggregation	Tanaka et al., 2004
	CK2	SH-SY5Y cells	No toxicity detected	S129A decreases inclusion formation while pS129 levels correlates with inclusion formation	Smith et al., 2005
	CK2 and other unidentified kinases	SH-SY5Y cells	S129D is toxic; S129A does not affect aSyn toxicity	increased pS129 was not attend with increased insoluble aggregates	Chau et al., 2009
	CK2	3D5 neuroblastoma cell line	–	pS129 promotes aSyn oligomerization and inclusion formation	Takahashi et al., 2007
	CK1, CK2	*In vitro* biochemical assay/SH-SY5Y cells/ transgenic mice M20 and M83	–	pS129 reduced recombinant aSyn fibril formation	Waxman and Giasson, 2008
	–	Mouse MN9D dopaminergic cells coexpressing human aSyn WT or S129D	S129D is protective	S129D promotes aSyn fibril or inclusion formation	Wu et al., 2011a
	GRK2, GRK5, PLK2, PLK3	Human brain neuroglioma H4 cell line	–	S129A increases inclusion formation	Gonçalves and Outeiro, 2013
	CK2 and PLKs	Rat oligodendroglial cell line OLN-93 coexpressing human p25aand aSyn WT or S129A/D	pS129 increases microtubule retraction followed by apoptosis and cell dead; S129A is protective while S129D behaves as WT, whoever with a smooth phenotype	pS129 promotes aSyn oligomers formation while S129A mutagenesis or CK2 and PLKs kinase inhibitors prevent it	Kragh et al., 2009
	PLK1, PLK2, PLK3	HEK293T/HeLa cells/ primary rat Neurons/ (Thy1)-h[A30P] aSyn transgenic mice	–	–	Mbefo et al., 2010
	Gprk2 (Grk2 *Drosophila* homolog)	*Drosophila*	Increased pS129 is toxic; S129D is toxic; S129A is protective	pS129 increases soluble oligomers formation but has no effect on inclusion formation	Chen and Feany, 2005; Chen et al., 2009
	–	SH-SY5Y cells/ transgenic*C. elegans*	S129D is protective while S129A is toxic	No insoluble oligomers or bigger aggregates were observed	Kuwahara et al., 2012
	–	Rat model involving viral delivery of WT or S129D/A aSyn	S129A is toxic while S129D is protective	S129D promotes inclusion formation while S129A reduce it	Gorbatyuk et al., 2008
	–	Rat model involving viral delivery of WT or A30P aSyn with S129D/A mutations	S129A is toxic while S129D has no effect	S129A increases aggregates formation while S129D forms fewer but larger aggregates	Azeredo Da Silveira et al., 2009
	CK1, CK2, PLK1, PLK2, PLK3	*In vitro* biochemical assay / QBI293 cells transfected with WT aSyn and treated with recombinant aSyn fibrils to induce the formation of aggregates, treated with kinases inhibitors or co-expressing kinases	–	Results obtained with different kinases suggest that phosphorylation of aSyn is independent of aSyn aggregate formation	Waxman and Giasson, 2011
	PLK2	HEK239T cells co transfected with aSyn and WT PLK2 or the kinase dead mutant (DM) PLK2; treated or not with PLK2 inhibitor / Rat model involving viral delivery of aSyn with either PLK2 WT or KDM	Increased pS129 aSyn by PLK2 reduces aSyn accumulation, suppresses dopaminergic neurodegeneration, and reverses hemiparkinsonian motor impairments by promoting aSyn autophagic clearence	–	Oueslati et al., 2013
Y133	Syk	*In vitro* biochemical assay/SH-N-BE and CHO cells	–	Syk-mediated aSyn phosphorylation decreases oligomerization	Negro et al., 2002
Y136	Syk	*In vitro* biochemical assay/SH-N-BE and CHO cells	–	Syk-mediated aSyn phosphorylation decreases oligomerization	Negro et al., 2002

The genetic mutant that attempts to mimic pS129 (S129D) aSyn was initially associated with pathology in a transgenic *Drosophila* model (Chen and Feany, 2005; Chen et al., 2009) while aSyn hyperphosphorylation and insolubility were correlated with the disease in transgenic mouse models of PD (Kahle et al., 2002; Freichel et al., 2007). However, opposite results were obtained in yeast, rat and *Caenorhabditis elegans* models of PD. Namely, S129E had no effect while the mutation S129A increased aSyn toxicity in budding yeast (Fiske et al., 2011; Sancenon et al., 2012). Moreover, in rat models using retrovirus-mediated expression of aSyn in neurons of the *substantia nigra*, the S129A variant also showed toxicity while the results for S129D were variable, showing either protecting (Gorbatyuk et al., 2008) or no effect (Azeredo Da Silveira et al., 2009). In *C. elegans* models, S129D aSyn was also protective, reducing neuronal dysfunction, while S129A expression resulted in severe motor dysfunction, growth retardation, and synaptic abnormality by lowering its membrane interaction (Kuwahara et al., 2012).

Similarly, while some reports suggest that pS129 promotes inclusion formation (Fujiwara et al., 2002; Smith et al., 2005; Arawaka et al., 2006; Takahashi et al., 2007; Gorbatyuk et al., 2008; Kragh et al., 2009; Wu et al., 2011a), others suggest that phosphorylation prevents or has no effect on inclusion formation (Lee et al., 2004a; Chen and Feany, 2005; Paleologou et al., 2008; Waxman and Giasson, 2008; Azeredo Da Silveira et al., 2009; Chau et al., 2009; Chen et al., 2009; Fiske et al., 2011; Sancenon et al., 2012) (Table 1).

*In vitro* biochemical studies also lead to conflicting results regarding the correlation between pS129 and fibrillization of aSyn. While S129 aSyn phosphorylated by casein kinase (CK)2 was found to form fibrils more readily than unphosphorylated aSyn *in vitro* (Fujiwara et al., 2002), different studies observed that fibrillization of aSyn is inhibited in purified pS129 S87A aSyn (where phosphorylation at S87 is blocked) (Paleologou et al., 2008; Waxman and Giasson, 2008), while a more recent study reposted that pS129 aSyn by polo-like kinase (PLK) 2 displays comparable fibrillization kinetics to the WT protein *in vitro* (Schreurs et al., 2014).

Studies performed in different cell and animal models are also not consensual regarding the correlation between aSyn pS129 and aggregation. Most studies performed in cell lines associate aSyn pS129 with increased formation of soluble oligomers (Arawaka et al., 2006; Kragh et al., 2009), cytoplasmic and nuclei aggregates (Arawaka et al., 2006; Wu et al., 2011a), and cytoplasmic inclusions (Smith et al., 2005; Takahashi et al., 2007) (Table 1). Toxicity was evaluated only in some of these studies and was interrelated with increased aggregation in one study (Kragh et al., 2009) but was found to be protective in another (Wu et al., 2011a).

In yeast cells, S129A aSyn is more toxic and forms more inclusions and oligomeric species of higher molecular weight than S129E or WT forms of aSyn (Sancenon et al., 2012; Tenreiro et al., 2014). Consistently, higher toxicity of the S129A variant is also associated with an increase in the generation of small, more soluble aggregates in rats (Azeredo Da Silveira et al., 2009).

To explain the discrepancies between the results obtained in different cell and animal models, several ideas have been put forward. One possibility is that the predominance of pS129 aSyn in LBs is not caused by its inherent propensity to aggregate but could be more related to the presence or absence of additional factors in the different models employed. Namely, the distinct results obtained in rat models could be eventually associated to dose-dependent interactions between rat aSyn and virally expressed mutant human aSyn, altering the aggregation properties of the protein, as has been demonstrated *in vitro* for mixtures of mouse and human aSyn (Rochet et al., 2000). On the other hand, in SH-SY5Y cells, co-expression of aSyn S129A with synphilin-1, an aSyn interacting protein that is also present in LBs, resulted in the formation of fewer inclusions than WT aSyn (Smith et al., 2005). In a *Drosophila* model, the obvious differences in the complexity of the nervous system and the absence of an aSyn homolog might explain the differences observed (Goedert, 2001; Hamilton, 2004).

Recently, it was also suggested that the discrepancies observed in the various studies might be due to different efficiencies of the different kinases in phosphorylating either S129 or other residues, as well as their differential pattern of expression in the different models (Oueslati et al., 2013; Schreurs et al., 2014). The conflicting results might also be due to differences in the dephosphorylation machinery involved in the dephosphorylation of aSyn, a process that is still understudied.

Another hypothesis is that phosphorylation could be an indirect cause of aSyn pathology, namely due to the impairment of the proteolytic machinery (Azeredo Da Silveira et al., 2009). There are several examples of proteins where phosphorylation works as a signal for protein degradation. If this is also the case for aSyn, then phosphorylated aSyn could accumulate in LBs due to proteasomal impairment (McNaught and Jenner, 2001; Shimura et al., 2001; Tanaka et al., 2001; Snyder et al., 2003; Grunblatt et al., 2004) leading to its accumulation and consequent aggregation.

Phosphorylation of aSyn in inclusions may be partially due to the intrinsic properties of aggregated aSyn to act as substrate for kinases but not phosphatases, as indicated by *in vitro* studies, suggesting that fibril and inclusion formation occur prior to phosphorylation and that this modification becomes more pronounced with disease progression (Waxman and Giasson, 2008; Mbefo et al., 2010; Paleologou et al., 2010; Waxman and Giasson, 2011).

In addition to S129, there are other phosphorylation sites in aSyn that may be relevant to aggregation and toxicity in synucleinopathies. This could either be due to a direct effect of the phosphorylation, or due to an effect on the cross-talk that likely occurs between phosphorylated states of these different residues. For example, phosphorylation of tyrosine residues Y125, Y133, and Y136 in the C-terminal segment of aSyn suppresses eosin-induced oligomerization (Negro et al., 2002). Phosphorylation at Y125 (pY125) has opposing effects to phosphorylation of S129 on aSyn neurotoxicity and soluble oligomer formation in a transgenic *Drosophila* model (Chen et al., 2009). Although pY125 does not directly affect the pS129 or vice versa, tyrosine phosphorylation is possibly acting downstream of pS129, as increasing pY125 levels rescued the neurotoxicity of a phospho-mutant S129D (Chen et al., 2009). This could be easily explained considering that different kinases are involved in the two phosphorylation events, which in turn can have behind completely different regulation pathways and physiological roles. Phosphorylation at Y125 diminishes with aging and is reduced in cortical tissue of DLB patients indicating a neuroprotective role (Chen et al., 2009). However, another recent study did not observe any significant differences in the levels of pY125 between PD brains and controls (Mahul-Mellier et al., 2014). In fact, phosphorylation at this residue was not detected in LBs of patients with DLB, in PD patients carrying the A53T mutation, nor in MSA cases (Anderson et al., 2006). This might be due to an increased sensitivity of Y125 to be dephosphorylated post mortem (Chen et al., 2009). Additionally, it could not be completely excluded that the observed effect on reduced oligomerization and concomitant toxicity was exclusively due to pY125, as the degree of Y133 and Y136 phosphorylation was not evaluated in this study performed in a *Drosophila* PD model (Chen et al., 2009). In fact, studies using recombinant aSyn demonstrated that the single phosphorylation of Y125 by Lyn and Fgr kinases does not affect oligomerization while the phosphorylation of all residues Y125, Y133 and Y136 by Syk prevents it (Negro et al., 2002). Recently, a new residue was detected as being phosphorylated in human brain tissues, the Y39, but without significant differences in the levels of pY39 between PD brains and controls (Mahul-Mellier et al., 2014). Importantly, in this same study, phosphorylation at Y39 and Y125 was found to play an important role in regulating aSyn clearance through proteasome and autophagy pathways (Mahul-Mellier et al., 2014).

S87 is, in addition to Y39, the only other residue outside the C-terminal region reported to undergo phosphorylation (pS87) *in vivo* (Paleologou et al., 2010). pS87 was found to be increased in brains of rat and mice models of synucleinopathies as well as in human brains from AD, LBD, and MSA patients (Paleologou et al., 2010) contradicting previous studies where phosphorylation of aSyn at this residue was not detected in either human brain samples or a transgenic mouse model of synucleinopathies (Fujiwara et al., 2002; Anderson et al., 2006; Waxman and Giasson, 2008). Again, results obtained using different systems were contradicting. pS87 may promote aSyn inclusion formation and decrease cell viability in SH-SY5Y and H19-7 cell lines (Kim et al., 2006). On the other hand, *in vitro* phosphorylation at this site inhibits aSyn fibril formation (Waxman and Giasson, 2008; Paleologou et al., 2010). Moreover, immunofluorescence staining of LBs isolated from fresh human brains using a specific anti-pS87 antibody allowed its detection and suggested that this phosphorylation occurs throughout the life span of LB development (Paleologou et al., 2010). More recently, S87E was found to inhibit aggregation and to protect against aSyn induced toxicity *in vivo*, namely by reducing aSyn aggregates, dystrophic fibers, and motor impairment in a rat model of PD where viral delivery was used to overexpress WT, S87A, and S87E aSyn in the substantia nigra (Oueslati et al., 2012).

Another aspect that might explain the discrepancies observed in the different studies is the employment of aSyn phosphorylation mutants. While several studies reported consistent results using in parallel genetic or pharmacological methods to alter aSyn phosphorylation status (Chen and Feany, 2005; Smith et al., 2005; Chen et al., 2009), other studies indicate that phospho-mutants may not fully recapitulate the real phosphorylation/unphosphorylation states of aSyn (Paleologou et al., 2008; Schreurs et al., 2014). In particular, it was shown that S129A and S129D/E mutations themselves could have effects on aSyn aggregation properties independent of their effects on phosphorylation, with the S129A mutation stimulating fibril formation while S129D/E mutations do not reproduce the effect of phosphorylation on the structural and aggregation properties of aSyn *in vitro* (Paleologou et al., 2008). However, in yeast we observed that S129G and S129A mutations, both blocking aSyn phosphorylation, were more toxic and resulted in increased inclusion formation excluding that the observed phenotypes were due to specific structural consequences of S129A mutation on aSyn (Tenreiro et al., 2014). Moreover, in this yeast model, S129E aSyn exhibited the same phenotype of toxicity and inclusion formation as the WT protein that is strongly phosphorylated on S129 by endogenous kinases (Tenreiro et al., 2014). Despite the inherent caveats, the use of aSyn phospho-mutants still remains as a unique and powerful means to interrogate the effects of phosphorylation. Regarding the use of phosphomimic mutants of other residues, S87E was shown to behave as pS87, at least with respect to its effects on aSyn aggregation, while the S87A mutant exhibited similar secondary structure and similar membrane binding and aggregation properties as the WT protein (Waxman and Giasson, 2008; Paleologou et al., 2010; Oueslati et al., 2012).

Mutants that abolish phosphorylation at tyrosine residues of aSyn (by replacing tyrosine by phenylalanine residues) were used in several *in vitro* and *in vivo* studies (Chen et al., 2009; Mahul-Mellier et al., 2014). However, there are no mutants that mimic the phosphorylated state of a tyrosine residue, restricting the use of mutants that attempt to mimic tyrosine phosphorylation.

### Physiological and pathological implications of aSyn phosphorylation

Initial studies suggested aSyn might be predominantly unphosphorylated under physiological conditions (Okochi et al., 2000; Fujiwara et al., 2002). It was hypothesized that changes in aSyn phosphorylation could represent a response to biochemical events associated with PD pathogenesis. Among these, mitochondrial complex I dysfunction, oxidative stress and proteasome dysfunction are processes that are known to be involved in synucleinopathies (Lee and Trojanowski, 2006; Lashuel et al., 2013). Increased levels of pS129 aSyn were observed upon proteasome inhibition or oxidative stress in SH-SY5Y cells over-expressing aSyn. In the case of proteasomal impairment, this seems to result in pS129 aSyn accumulation through an increase in the activity of the kinase(s) involved, a decrease in protein turnover and, ultimately, in increased cell death (Waxman and Giasson, 2008; Chau et al., 2009). The kinases involved were not fully characterized but CK2 was found to be one of them. On the other hand, it is known that aggregation of aSyn itself leads to proteasome impairment (Tanaka et al., 2001; Snyder et al., 2003; Lindersson et al., 2004), which in turn could lead to CK2 activation and eventually to increased levels of pS129. It is important to note that phosphorylation of S129 appears not to be a general response to cellular stress, as inhibition of complex I had little effect on pS129 aSyn levels (Waxman and Giasson, 2008; Chau et al., 2009).

The low levels of pS129 aSyn under physiological conditions as well as the absence of other phosphorylated residues such as pY39, pS87 and pY125 (Okochi et al., 2000; Fujiwara et al., 2002; Anderson et al., 2006) could also be related to a faster degradation of this form under normal conditions. In fact, the phosphorylation status of aSyn was recently correlated with clearance mechanisms (Oueslati et al., 2013; Mahul-Mellier et al., 2014). Namely, blocking S129 phosphorylation in a yeast model lead to impaired aSyn clearance by autophagy (Tenreiro et al., 2014). In line with this observation increased levels of pS129 by overexpression of PLK2 suppress dopaminergic neurodegeneration, and reverse hemiparkinsonian motor impairments in a rat model of PD by promoting aSyn autophagic degradation (Oueslati et al., 2013). Moreover, phosphorylation at Y39 and Y125 by c-Abl kinase protects aSyn against its degradation via the autophagy and proteasome pathways in cortical neurons (Mahul-Mellier et al., 2014).

Phosphorylation also seems to alter the subcellular localization of aSyn. While pS129 aSyn was found to be preferentially localized in the nuclei of dopaminergic neurons in rat and mouse models of synucleinopathy (Yamada et al., 2004; Wakamatsu et al., 2007), in other studies using PD rat models the phospho-resistant S129A was found to be localized in the nucleus at higher levels than the S129D form, and was found to correlate with enhanced toxicity (Gorbatyuk et al., 2008; Azeredo Da Silveira et al., 2009). Our group demonstrated that S129 phosphorylation modulates the shuttling of aSyn between nucleus and cytoplasm in human neuroglioma cells, using photoactivatable green fluorescent protein as a reporter. Moreover, we also found that co-expression of aSyn with different kinases altered the translocation dynamics of the protein. While G protein-coupled receptor kinase 5 (GRK5) promotes the nuclear localization of aSyn, PLK2 and 3 modulate the shuttling of the protein between the nucleus and cytoplasm (Gonçalves and Outeiro, 2013). This difference might reflect different aSyn phosphorylation patterns in S129 and/or other residues, or phosphorylation of other targets besides aSyn. Very recently, G51D aSyn was found to exhibit enhanced nuclear localization and to be hyperphosphorylated on S129 in primary neurons (Fares et al., 2014). Although the function of aSyn in the nucleus is still unclear, it seems this is related with a pathological role that is independent of aSyn aggregation. In particular, nuclear localization of aSyn increases under oxidative stress conditions (Xu et al., 2006; Monti et al., 2010; Siddiqui et al., 2012). Nuclear aSyn interacts with histones, inhibits acetylation and promotes neurotoxicity (Goers et al., 2003; Kontopoulos et al., 2006). Moreover, aSyn might act as a transcriptional regulator, binding promoters such as PGC1-alpha, a master regulator of mitochondrial gene expression (Siddiqui et al., 2012).

Phosphorylation at S129 reduces the affinity of aSyn for lipids (Okochi et al., 2000; Pronin et al., 2000; Fujiwara et al., 2002; Yamada et al., 2004). Also pS87 was described to significantly reduce aSyn binding to lipid vesicles (Paleologou et al., 2010). Therefore, the phosphorylation status of aSyn might regulate its role in synaptic vesicle dynamics in physiological conditions and might contribute to its pathological role in abnormal dopamine neurotransmission (Lundblad et al., 2012; Scott and Roy, 2012).

It was also reported that aSyn inhibits tyrosine hydroxylase activity, a rate-limiting enzyme in dopamine biosynthesis, in dopaminergic MN9D cells, while the phosphomimic mutant of aSyn, S129D, relieves this inhibition and results in an increase of dopamine content in cells (Wu et al., 2011a). Recently, it was also observed that membrane-associated aSyn enhances dopamine uptake capacity in dopaminergic SH-SY5Y cells by the dopamine transporter through GRKs-mediated S129 phosphorylation (Hara et al., 2013).

The phosphorylation status of aSyn could also modulate its protein-protein interactions. The unphosphorylated form of S129 associates mainly with mitochondrial electron transport proteins while pS129 associates with cytoskeletal, vesicular trafficking proteins and enzymes involved in protein serine phosphorylation (McFarland et al., 2008). Phosphorylation also appears to have an important role in the regulation of aSyn axonal transport as the S129D mutation significantly reduces its rate of transport in neurons, likely due to the modulation of the interaction of aSyn with motor and/or accessory proteins involved in this process (Saha et al., 2004). Moreover, the interplay between the different phosphorylated residues could also contribute to increase the diversity in the possible protein interactors. In fact, several differences were observed in the set of proteins that were found to interact with S129 or Y125-phosphorylated forms of aSyn (McFarland et al., 2008). Both, S129 and Y125 residues are localized in the C-terminal region of aSyn which has been implicated in the majority of aSyn interactions with proteins (Jensen et al., 1999; Giasson et al., 2003a; Fernandez et al., 2004) reinforcing the relevance that phosphorylation in these residues could modulate the biological role of aSyn.

The C-terminus of the protein was also implicated in aSyn interactions with metal ions (Paik et al., 1999; Brown, 2007). These interactions influence the structure and propensity for aggregation of aSyn *in vitro* and in cell culture models of synucleinopathies (Paik et al., 1999; Brown, 2007; Wright et al., 2009). Interestingly, a recent study showed that pY125 and pS129 alter the binding sites of metal ions and increase the binding affinity of Cu(II), Pb(II), and Fe(II), but not Fe(III), a feature that could modulate aSyn function as well as aggregation (Lu et al., 2011).

Phosphorylation at S87 could also modulate protein-protein interactions, as some proteins were found to interact via the non-amyloid component (NAC) region, in which this residue is located. As an example, it is possible that S87 phosphorylation alters the interaction with phospholipase D (PLD) 2, an enzyme involved in lipid-mediated signaling cascades and vesicle trafficking (Outeiro and Lindquist, 2003; Payton et al., 2004).

Fyn and Src kinases are able to phosphorylate aSyn at Y125, suggesting phosphorylation in this residue might also modulate spatial learning and synaptic plasticity, due to the role these kinases play in these processes (Zhao et al., 2000). On the other hand, Fyn and Src are non-receptor-type PTKs activated by extracellular factors like neurotrophic factors and growth factors, suggesting that the phosphorylation state of aSyn can be regulated by extracellular signaling molecules, such as neurotrophins, cytokines, and cell adhesion molecules (Nakamura et al., 2001). Recently, a new connection between tyrosine phosphorylation of aSyn and synaptic plasticity was established with the identification of Y39 as the main target of phosphorylation by c-Abl protein tyrosine kinase (Mahul-Mellier et al., 2014), a kinase that plays an important role in the development of the central nervous system (CNS) and in neuronal plasticity (Moresco and Koleske, 2003; Moresco et al., 2003). Interestingly, c-Abl is upregulated in PD brains (Ko et al., 2010; Hebron et al., 2013a) as well as in other neurodegenerative diseases (Schlatterer et al., 2011). This tyrosine kinase is activated by increased levels of aSyn and, in turn, increased c-Abl activity leads to aSyn accumulation (Hebron et al., 2013a,b) by increasing pY39 and, to a lesser extent pY125, thereby affecting clearance pathways (Mahul-Mellier et al., 2014). Thus, phosphorylation of aSyn at tyrosine residues could be relevant in the context of the alterations of synaptic functions observed in PD and other synucleinopathies.

It remains unclear how familial mutations of aSyn might alter the phosphorylation of the protein, but it is likely that different mutations may influence phosphorylation in different residues. In transgenic mice expressing either E46K or A53T aSyn, inclusions were found to be strongly phosphorylated at S129 (Emmer et al., 2011). In HEK cells, S129 phosphorylation by GRK6 or PLK2 is equally efficient in WT or in G51D aSyn, although pS129 enhanced nuclear localization of G51D compared to WT aSyn (Fares et al., 2014). Moreover, while the A53T mutant shows similar phosphorylation levels to WT aSyn in SH-SY5Y cells (Smith et al., 2005), and slower *in vitro* phosphorylation kinetics by CK2 (Ishii et al., 2007), it was observed that detergent-insoluble aSyn from patients carrying the A53T mutation was hyper-phosphorylated at S129 (Anderson et al., 2006). In any case, additional studies on the interplay between aSyn mutations and phosphorylation are needed.

### Kinases involved in aSyn phosphorylation

Several kinases have been implicated in aSyn phosphorylation (Table 1). S129 can be phosphorylated by G-protein coupled receptor kinases (GRK1, GRK2, GRK5 and GRK6) (Pronin et al., 2000; Arawaka et al., 2006; Sakamoto et al., 2009), casein kinases 1 and 2 (CK1, CK2) (Okochi et al., 2000; Smith et al., 2005; Ishii et al., 2007; Takahashi et al., 2007; Wakamatsu et al., 2007; Waxman and Giasson, 2008; Zabrocki et al., 2008), and the polo-like kinases (PLKs) (Inglis et al., 2009). The leucine-rich repeat kinase 2 (LRRK2) was also shown to phosphorylate aSyn at S129 (Qing et al., 2009), but this remains highly controversial as no other studies were able to confirm this, despite the existence of a clear interaction between the two proteins (Qing et al., 2009; Guerreiro et al., 2013).

Recent studies revealed that, in addition to phosphorylating agonist-occupied G protein-coupled receptors (GPCRs), GRKs may also phosphorylate non-receptor substrates, including the four members of the synuclein family (aSyn, beta-, gamma-synuclein and synoretin) (Pronin et al., 2000). Overexpression of GRK2 or GRK5 in COS-1 cells showed that these kinases phosphorylate aSyn at S129 (Pronin et al., 2000). Phosphorylation of aSyn at S129 by endogenous GRKs was also demonstrated in HEK293 cells and it was observed that GRK3 and GRK6 play the main role in this modification (Sakamoto et al., 2009).

GRK5 was found to colocalize with aSyn in the LBs of the substantia nigra of PD patients, but was not detected in cortical LBs of DLB, or in the glial cytoplasmic inclusions of MSA (Arawaka et al., 2006). Overexpression of aSyn increased GRK5 protein expression in both, SH-SY5Y cells and in brain extracts of transgenic mice expressing human aSyn (Liu et al., 2010). A genetic association study performed in the Japanese population revealed a haplotypic association of the GRK5 gene with susceptibility to sporadic PD (Arawaka et al., 2006). However, another genetic association study performed in Southern Italy failed to correlate GRK5 polymorphisms with sporadic PD (Tarantino et al., 2011). The knockdown of endogenous GRK5 in SH-SY5Y cells fails to suppress phosphorylation of aSyn (Liu et al., 2010) confirming the involvement of other kinases in this phosphorylation.

CK1 and CK2 also phosphorylate aSyn at S129 in yeast (Zabrocki et al., 2008), in mammalian cells (Okochi et al., 2000; Waxman and Giasson, 2008) and in rat primary cortical neurons (Ishii et al., 2007).

CK1-mediated phosphorylation at S129 may counteract aSyn toxicity by attenuating vesicular trafficking defects and restoring synaptic transmission in some extension (Sancenon et al., 2012). However, higher levels of aSyn could result in protein mislocalization in other compartments, ultimately leading to defects in synaptic vesicle homeostasis and neurotransmission. In addition, excess aSyn may form inclusions that sequester CK1, depleting CK1 activity and exacerbating synaptic defects, generating a toxic vicious cycle. CK1 was also found to phosphorylate aSyn at S87 (Okochi et al., 2000), and to colocalize with pS87 in transgenic mice and in LB-like structures in LBD/PD diseased brains (Paleologou et al., 2010). β subunits of CK2 were also found to colocalize with LBs in PD brains (Ryu et al., 2008). Interestingly, oxidative stress imposed by iron overload causes upregulation of CK2 which, in turn, leads to increased pS129 aSyn with a concomitant increase in oligomerization and inclusion formation (Takahashi et al., 2007). In SH-SY5Y cells, the increase in aSyn phosphorylation under oxidative stress is mediated by CK2 and correlates with enhancement of inclusion formation (Smith et al., 2005).

*In vitro* studies, employing kinase assays, showed that PLK1, PLK2 and PLK3 are also capable of phosphorylating aSyn at S129 (Inglis et al., 2009; Mbefo et al., 2010). The PLKs comprise a family of conserved Ser/Thr protein kinases that are known to play critical roles on cell cycle regulation, cellular response to stress and carcinogenesis (Ng et al., 2006). PLK2 and PLK3 are expressed in response to synaptic activation and appear to be involved in synaptic plasticity, remodeling and homeostasis (Kauselmann et al., 1999; Seeburg et al., 2005, 2008), suggesting these kinases could be important actors in modulating the normal physiology of aSyn.

PLK2 and PLK3 partially colocalize with pS129 aSyn in primary hippocampal neurons as well as in cortical brain areas of aSyn transgenic mice, reinforcing the idea that S129 phosphorylation by the PLKs might also occur in human brain (Mbefo et al., 2010). Consistently, PLK2 levels are elevated in brains of patients with AD and DLB, and correlate with the increased levels of pS129 aSyn, further supporting a role for this kinase in disease (Mbefo et al., 2010). PLK2 is also involved in the phosphorylation of aggregated aSyn *in vitro* (Mbefo et al., 2010) and in cell culture (Waxman and Giasson, 2011). A prominent role of PLK2 as a regulator of aSyn turnover was recently described (Oueslati et al., 2013). Importantly, PLK2-mediated pphosphorylation at S129 of aSyn is protective in a rat model of PD, by promoting aSyn autophagic degradation (Oueslati et al., 2013). Recently, we also described that PLK2 mediates aSyn inclusion formation in yeast and in mammalian cells by a S129 phosphorylation-independent mechanism (Basso et al., 2013).

Only CK1 and the dual specificity tyrosine regulated kinase 1A (Dyrk1A) were found to phosphorylate aSyn at S87, and this was based on *in vitro* kinase assays and cells culture models (Okochi et al., 2000; Waxman and Giasson, 2008). CK1 colocalizes with pS87 in neuronal inclusions in a PD mouse model and in LB-like structures in LBD/PD diseased brains (Paleologou et al., 2010).

Several tyrosine kinases phosphorylate aSyn. The Y125 residue is target of phosphorylation by Fyn (Nakamura et al., 2001), Syk (Negro et al., 2002), Lyn (Negro et al., 2002), c-Frg (Negro et al., 2002), Src (Ellis et al., 2001) and c-Abl (Mahul-Mellier et al., 2014). Syk also phosphorylates Y133 and Y136 (Negro et al., 2002), and c-Abl also phosphorylates Y39 aSyn (Mahul-Mellier et al., 2014).

An emerging concept is that certain phosphorylation events might promote or prevent subsequent phosphorylation events in other residues (Negro et al., 2002; Mbefo et al., 2010). In fact, the double mutation of Y133 and Y136 to phenylalanines, designed to prevent phosphorylation in these residues, augments Y125 phosphorylation by Lyn (Negro et al., 2002). Phosphorylation or binding of c-Abl at Y125 was also found to decrease the propensity of this kinase to phosphorylate aSyn at Y39 (Mahul-Mellier et al., 2014).

## Tau phosphorylation in alzheimer's disease and other tauopathies

The “amyloid cascade hypothesis” was formulated after an amyloid precursor protein (APP) mutation was reported in a family with AD-typical histology and proposes that accumulation of an APP cleavage product, beta amyloid (Aβ), induces the biochemical, histologic, and clinical changes AD patients manifest (Hardy and Higgins, 1992). Later, Aβ oligomers were suggested to trigger neurotoxicity in AD probably via tau phosphorylation. Glycogen synthase kinase-3β (GSK-3β) activation was proposed as mediator of Aβ42 oligomer-induced effects on tau phosphorylation in P301L mice (Selenica et al., 2013).

### The role of phosphorylation on tau cytotoxicity and aggregation

Tau, in its longest isoform, contains 35 threonine, 45 serine, and 5 tyrosine residues meaning that nearly 20% of the tau protein has the potential to be phosphorylated. Early studies revealed that tau is more efficient at promoting microtubules (MT) assembly in a more unphosphorylated state (Lindwall and Cole, 1984). A few years later, tau was demonstrated to make up the paired-helical filaments (PHFs) which form the neurofibrillary tangles (NFTs) found in AD brain and to be abnormally phosphorylated in these structures (Grundke-Iqbal et al., 1986; Goedert et al., 1988; Kosik et al., 1988; Wischik et al., 1988). Further analyses revealed that PHF-tau is phosphorylated at “pathological” sites, which was assumed to contribute to pathological processes in AD. Enhanced immunoreactivity in human AD tissue was observed with the phosphorylation-dependent antibodies AT8 (epitope pS199/pS202/pT205), PHF-1 (epitope pS396/pS404), and pS262 (Gu et al., 2013a; Mondragon-Rodriguez et al., 2014). Hyperphosphorylation of tau was shown to be involved in tau aggregation and cytotoxicity (Table 2) (Kosik and Shimura, 2005; Noble et al., 2013).

**Table 2:** **Tau phosphorylation sites and effects**.

**Tau residue**	**Kinase**	**Model**	**Cytotoxicity**	**Aggregation**	**References**
S199/S202/T205 (AT8, CP13 epitopes)	–	*In vitro* biochemical assay	–	S199E/S202E/T205E affects MT binding, MT polymerization and aggregation of tau	Sun and Gamblin, 2009; Bibow et al., 2011
	GSK-3β	*In vitro* biochemical assay	–	Pre-assembled pS199/pT205 tau filaments form large tangle-like structures	Rankin et al., 2008
	–	PC12 cells	S199E/S202E/T205E cause expansion of the space between MTs and inhibit mitochondrial movement in neurites and axons	–	Shahpasand et al., 2012
	GSK-3β	Rat hippocampal slices	NMDA receptor activation induces pS199/pS202 and facilitates LTD induction	–	Mondragon-Rodriguez et al., 2012
	–	rTg4510 tau transgenic mice	O-linked N-acetylglucosamine modification (O-GlcNAcylation) of tau lessens pS202/pT205, reduces the number of dystrophic neurons	O-GlcNAcylation of tau protects against tau aggregation	Graham et al., 2013
	–	TPR50 tau transgenic mice	pS202/pT205 increased with age, MT hyperdynamics, impaired axonal transport, cognitive deficits earlier than aggregates	Tau insolubility and intracellular accumulation	Onishi et al., 2014
	GSK-3β	pR5 tau transgenic mice	–	Increased pS202/pT205 is associated with fibrillar tau pathology	Kohler et al., 2013
	Cdk5	P25/Cdk5 transgenic mice	–	Increased pS202/pT205 is associated with aggregated tau filaments	Cruz et al., 2003
	–	TauE391 truncated transgenic mice	–	Truncation at E391 increases pS202/pT205; tau accumulation, mislocalization, tangle formation	McMillan et al., 2011
	Cdk5, GSK-3β	P25/P301L transgenic mice	–	Increased pS202 is associated with increased number of NFTs	Noble et al., 2003
	LRRK2	LRRK2/TauP301L transgenic mice	–	LRRK2 expression increases pS199/pS202/pT205 of insoluble tau	Bailey et al., 2013
	–	rTg4510 tau transgenic mice	–	pS202/pT205 in TBS-extractable tau which consists of granular aggregates and short filaments	Sahara et al., 2013
	–	IHC on paraffin- sections AD brain	–	Enhanced pS199/pS202/pT205 in mature NFTs	Mondragon-Rodriguez et al., 2014
	–	homogenates from AD brain tissue, AD synaptosomes	–	Oligomers positive for pS202/pT205 accumulate at synapses in AD	Henkins et al., 2012; Lasagna-Reeves et al., 2012; Tai et al., 2012
S262/S356 (12E8 epitope)	MARK2	*In vitro* biochemical assay	–	Acetylation on S262/S356 inhibits its phosphorylation and tau aggregation	Schwalbe et al., 2013; Cook et al., 2014
	MARK4	rat primary hippocampal neurons	Increased pS262/pS356 is associated with decrease in synaptic markers, loss of spines and synapses	–	Yu et al., 2012
	–	rTg4510 tau transgenic mice	O-GlcNAcylation of tau lessens pS262/pS356, reduces the number of dystrophic neurons	O-GlcNAcylation of tau protects against tau aggregation	Graham et al., 2013
	–	rTg4510 tau transgenic mice	–	pS262/pS356 in TBS-extractable tau which consists of granular aggregates and short filaments	Sahara et al., 2013
S262	DAPK1	HEK293,N2a cells	Tau expression antagonizes DAPK1 induced apoptosis with simultaneous pS262, no up-regulation of kinases	–	Duan et al., 2013
	PKA	Rat hippocampal neurons	pS262 mediates toxicity via MT instability; accelerated degradation of synaptophysin	–	Qureshi et al., 2013
	GSK-3β	Cortical neurons, rat hippocampal slices	Stress-induced increase of pS262 reduces cell viability	–	Selvatici et al., 2013
	Par-1	*Drosophila*	pS262 contributes to tau-mediated neurodegeneration	–	Iijima-Ando et al., 2012
	MARK2 MARK4	Paraffin sections AD brain	–	MARK-tau interactions and pS262 correlate with Braak stages	Gu et al., 2013a
T231/S235 (AT180,PHF-6 epitopes)	–	*In vitro* NMR measurements	–	pT231/pS235 has a helix stabilizing role, potentially affecting tau function and aggregation	Sibille et al., 2011
	DAPK1	HEK293,N2a cells	tau expression antagonizes DAPK1 induced apoptosis with simultaneous pT231, no up-regulation of kinases	–	Duan et al., 2013
	GSK-3β	Rat hippocampal slices	NMDA receptor activation induces pT231/pS235 and facilitates LTD induction	–	Mondragon-Rodriguez et al., 2012
	–	TauE391 truncated transgenic mice	–	Truncation of tau at E391 increases pT231/pS235, tau accumulation, mislocalization, and tangle formation	McMillan et al., 2011
	–	rTg4510 tau transgenic mice	–	pT231/pS235 in TBS-extractable tau which consists of granular aggregates and short filaments	Sahara et al., 2013
	GSK-3β	SAMP8 mice	GSK-3β antisense treatment decreases pT231/pS235; reduced oxidative stress, improved learning and memory	–	Farr et al., 2013
	–	Homogenates from AD brain tissue	–	Identification of pT231-positive oligomers at early AD stages	Lasagna-Reeves et al., 2012
S396/S404 (PHF-1, AD2, PHF-13 epitopes)	MARK2	*In vitro* biochemical assay	–	Acetylation on S396/S404 inhibits its phosphorylation and tau aggregation	Cook et al., 2014
	–	*In vitro* biochemical assay	–	S396E/S404E affects MT binding, MT polymerization and aggregation of tau	Sun and Gamblin, 2009; Bibow et al., 2011
	GSK-3β	*In vitro* biochemical assay	–	pre-assembled pS396/pS404 tau filaments form large tangle-like structures	Rankin et al., 2008
	LRRK2	*In vitro* biochemical assay, SH-SY5Y cells, LRRK2 tg mice	–	LRRK2 increases pS396, pT149, and pT153, and aggregation of tau	Bailey et al., 2013; Kawakami et al., 2014
	DAPK1	HEK293,N2a	Tau expression antagonizes DAPK1 induced apoptosis with simultaneous pS396, no up-regulation of kinases	–	Duan et al., 2013
	GSK-3β	Rat hippocampal slices	NMDA receptor activation induces pS396/pS404 and facilitates LTD induction	–	Mondragon-Rodriguez et al., 2012
	GSK-3β	Cortical neurons, rat hippocampal slices	Stress-induced increase of pS404 reduces cell viability	–	Selvatici et al., 2013
	–	rTg4510 tau transgenic mice	–	pS396/pS404 in TBS-extractable tau which consists of granular aggregates and short filaments	Sahara et al., 2013
	–	rTg4510 tau transgenic mice	O-GlcNAcylation of tau lessens pS396/pS404, reduces the number of dystrophic neurons	O-GlcNAcylation of tau protects against tau aggregation	Graham et al., 2013
	Cdk5, GSK-3β	p25/P301L transgenic mice	–	Increased pS396/pS404 is associated with increased number of NFTs	Noble et al., 2003
	Cdk5	p25/Cdk5 transgenic mice	–	Increased pS396/pS404 is associated with aggregated tau filaments	Cruz et al., 2003
	GSK-3β	P301L and GSK-3β /P301L transgenic mice	–	Increased pS396/pS404, increased tangle pathology but also longer survival than P301L mice.	Terwel et al., 2008
	–	AD material: homogenates, synaptosomes, paraffin sections	–	Oligomers positive for pS396 and/or pS404 accumulate at synapses in AD at different stages	Henkins et al., 2012; Lasagna-Reeves et al., 2012; Tai et al., 2012; Mondragon-Rodriguez et al., 2014
	–	IHC on paraffin sections AD brain	–	Content of tangles rather than phosphorylated tau lead to altered spine morphology and spine loss	Merino-Serrais et al., 2013
T212/S214/T217 (AT100 epitope)	–	*In vitro* biochemical assay	–	T212E/S214E/T217E affects MT binding, MT polymerization, and aggregation of tau	Bibow et al., 2011
	–	*C. elegans*	–	Inhibition of tau aggregation is paralleled by reduced pT212/pS214/pT217 and mitigates proteotoxicity	Fatouros et al., 2012
	GSK-3β	pR5 tau transgenic mice (P301L)	–	Increased pT212/pS214/pT217 is associated with fibrillar tau pathology	Kohler et al., 2013
	–	rTg4510 tau transgenic mice	–	pT212 in TBS-extractable tau which consists of granular aggregates and short filaments	Sahara et al., 2013
S422 residue	GSK-3β	pR5 tau transgenic mice	–	Increased pS422 is associated with fibrillar tau pathology	Kohler et al., 2013
	–	rTg4510 tau transgenic mice	–	pS422 in TBS-extractable tau which consists of granular aggregates and short filaments	Sahara et al., 2013
	–	AD synapses	–	Increased pS422 in AD synapses; SDS-stable tau oligomers and aggregates.	Henkins et al., 2012

Abnormal high levels of intracellular tau are frequently observed in AD patients and may be directly implicated in tau aggregation, PHF formation, and neuron loss (Gomez-Isla et al., 1997). It was speculated that the hyperphosphorylation of tau precedes NFT pathology and, more important, is a key event for the integration of tau into fibrils (Bancher et al., 1991). The staging of AD-related neurofibrillary pathology using a silver stain technique was revised using immunostaining for hyperphosphorylated tau at the AT8 epitope (Braak et al., 2006). Several studies addressed the question whether the pattern of tau hyperphosphorylation correlates with the progression of neuronal cytopathology and the formation of higher order tau species in AD. Brain tissue was classified into pre-NFTs, intra-neuronal NFTs and extra-neuronal NFTs, and was examined regarding the most prominent staining of phosphorylation-dependent tau antibodies. Epitopes that were associated with pretangle, non-fibrillar tau include pS199, pS202, pT231, pS262, pT153, and S409. Intraneuronal fibrillar structures were stained with antibodies recognizing pS46, pT175/pT181, pT231, pS262/pS356 (12E8 epitope), pS396, pS422, and pS214. Epitopes associated with extracellular filamentous tau include AT8, AT100 (pT212/pS214), and PHF-1 (Morishima-Kawashima et al., 1995b; Kimura et al., 1996; Augustinack et al., 2002). Notably, with progression of the disease, tau is phosphorylated at pathological multiple-site epitopes (AT8, AT100, AT180, PHF-1, 12E8). Tau inclusions were observed in other neurodegenerative disorders such as MSA (Giasson et al., 2003b), familial and sporadic PD (Ishizawa et al., 2003; Rajput et al., 2006), and in Down syndrome (Flament et al., 1990; Mondragon-Rodriguez et al., 2014). Elevated levels of AT180 (pT231/pS235)-phosphorylated tau were detected in the cerebrospinal fluid (CSF) of patients with mild cognitive impairment who later went on to develop AD (Arai et al., 2000a).

Several animal models were generated to recapitulate hyperphosphorylation of tau and the formation of NFTs as key aspects of tauopathies (Ribeiro et al., 2013). Some studies showed that the overexpression of human mutant tau in transgenic mice led to increased phosphorylation of tau and the formation of tau inclusions, aggregates, and fibrils. Phosphorylation of tau was detected at the well-known disease-related epitopes S202, T205, S212, S216, T231, S262, S356, S422, AT100 (Kohler et al., 2013; Nilsen et al., 2013; Sahara et al., 2013). Likewise, overexpression of LRRK2 or p25/Cyclin-dependent kinase-5 (Cdk5) in mice resulted in hyperphosphorylation of tau, tau aggregation into NFT-like structures, and neuronal death (Cruz et al., 2003; Noble et al., 2003; Bailey et al., 2013). Other models took advantage of the co-expression of other disease-associated proteins such as APP and presenilin 1 (Oddo et al., 2003; Grueninger et al., 2010), or made use of the injection of Aβ fibrils (Gotz et al., 2001).

Almost all currently available animal models in AD are based on the over-expression of pathogenic mutant tau forms. Therefore, it debatable how well these models recapitulate AD cases where there are no mutations in either tau or APP. However, the first models of tauopathy, based on the overexpression of either 3-repeat or 4-repeat human WT tau, presented tau hyperphosphorylation but no NFT formation. Expression of tau-P301L, often in conjunction with other disease-associated proteins, is the most widely used and most successful approach to recapitulate key aspects of AD such as tau hyperphosphorylation, aggregation, and filament formation as well as neuron death. In these models, it is often not clear what drives tau hyperphosphorylation. *In vitro* studies may help to decipher the impact of specific pathogenic mutations on tau phosphorylation but existing data are not consistent. The well-known FTDP-17-associated missense tau mutations R406W, V337M, G272V, and P301L were shown to make tau a more favorable substrate for phosphorylation by rat brain kinases, in comparison to WT tau protein (Alonso Adel et al., 2004). In another study, the same mutations were shown to promote or inhibit phosphorylation at specific sites (Han et al., 2009). *In vitro* phosphorylation by recombinant GSK-3b exerted reduced phosphorylation of the R406W mutation, probably through long-range conformational changes. Conversely, P301L and V337M mutations had no effect (Connell et al., 2001). Similar results were obtained in cell culture (Dayanandan et al., 1999). In contrast, several other studies using cell culture models and human brain tissue indicate that the R406W mutation reduces tau phosphorylation, not only at the neighboring PHF1 epitope but at several positions (Miyasaka et al., 2001; Deture et al., 2002; Tackenberg and Brandt, 2009; Gauthier-Kemper et al., 2011). However, depending on the cellular context, R406W was also shown to increase phosphorylation, and other mutations, such as V337M, reduced phosphorylation of tau at specific sites (Deture et al., 2002; Krishnamurthy and Johnson, 2004). Alterations in the phosphorylation state can have tremendous effects on the structural properties, function, and pathology of tau as discussed below.

*In vitro* data imply that phosphorylation of tau at certain epitopes directly impacts on local structural properties or the global conformation of tau which in turn may affect its assembly into PHFs. Different sites were suggested to be important for the aggregation propensity and filament formation of tau including AT8, AT100, AT180, PHF-1, and S305 upstream of the PHF6-hexapeptide motif which is known to be important for tau fibrillization (Sun and Gamblin, 2009; Bibow et al., 2011; Inoue et al., 2012). Some studies suggested that the compaction of the paperclip conformation of tau becomes tighter or looser depending on phosphorylation at the AT8, PHF-1, and AT100 epitopes (Jeganathan et al., 2008; Bibow et al., 2011). Likewise, phosphorylation within the repeat region, particularly at KXGS motifs, induced specific conformational changes that altered the MT binding properties of tau (Fischer et al., 2009). In other cases, structural changes were localized in the proximity of the phosphorylation sites without affecting the global conformation (Schwalbe et al., 2013).

Despite intensive research in the field, the contribution of phosphorylation to the formation of tau aggregates is still controversial (Table 2). Recent results from *in vitro* experiments, showing that recombinant unphosphorylated tau induced fibril formation similar to AD-derived PHFs, questioned the necessity of tau phosphorylation for the fibrillization process (Morozova et al., 2013). Furthermore, altered spine morphology and spine loss in tissue of AD cases were attributed to the content of tangles rather than to the amount of phosphorylated tau (Merino-Serrais et al., 2013). In a study using PS19 mice (tauP301S mutation), synthetic tau fibrils induced NFT pathology in the absence of tau hyperphosphorylation (Iba et al., 2013). The introduction of “pro-” and “anti-” aggregation mutations revealed that hexapeptide motifs of tau may function as a core to form local β-sheet structure and, subsequently, to induce PHF formation (Von Bergen et al., 2000; Eckermann et al., 2007). Enhanced tau levels, via stabilization of tau mRNA, may contribute to tau pathology independent of tau phosphorylation (Qian et al., 2013).

Other PTMs of tau might interfere with its phosphorylation, thereby influencing the structure, function and regulation of the protein, but the data are not consistent. KXGS motifs were found to be hypoacetylated and hyperphosphorylated in patients with AD, consistent with *in vitro* data showing that the acetylation of tau prevents its phosphorylation and inhibits tau aggregation (Irwin et al., 2013; Cook et al., 2014). In contrast, acetylation of tau at K280 was associated with phosphorylation at the AT8 epitope in tau aggregates of tau transgenic mice, and detected in post-mortem tissue of cases with AD or other tauopathies (Min et al., 2010; Cohen et al., 2011; Irwin et al., 2012). Recent evidence was provided that tau itself possesses acetyltransferase activity, and is capable of catalyzing self-acetylation (Cohen et al., 2013). *In vitro*, O-linked β-N-acetylglucosaminylation (O-GlcNAcylation) at S400 was inversely correlated with tau phosphorylation at S396 (Smet-Nocca et al., 2011). However, treatment of tau transgenic mice with an O-GlcNAcase inhibitor increased tau O-GlcNAcylation, hindered the formation of tau aggregates, and slowed neurodegeneration without affecting the phosphorylation of tau (Yuzwa et al., 2012; Graham et al., 2013).

Phosphorylation of tau at several residues mediates cellular toxicity (Table 2). Many data implicate that phosphorylation of tau needs to be well balanced. It was hypothesized that the detachment of tau from MTs results in impaired MT stability and excess amount of unbound hyperphosphorylated tau in the cytosol, thereby contributing to toxic insult. *In vitro* experiments provided evidence that phosphorylation of tau at S262, T231, and S214 is necessary for the full detachment of tau from MTs (Illenberger et al., 1998; Sengupta et al., 1998). Consistently, enhanced phosphorylation at S262 and T231 resulted in MT instability and cytotoxicity in cell and animal models (Steinhilb et al., 2007; Qureshi et al., 2013). Moreover, pathological processes were rescued by overexpression and activation of microtubule-affinity-regulating kinases (MARKs) that phosphorylate tau at KXGS motifs of the repeat domains (Mandelkow et al., 2004; Thies and Mandelkow, 2007). Aberrant phosphorylation of tau at pathological sites may result in altered tau-MT binding, thereby affecting the organization and dynamics of MT networks. This in turn may compromise axoplasmic flow and proper neuronal function, and ultimately cause cell death. The phosphorylation within KXGS motifs, especially at S262, and GSK-3β seem to take key roles among the phosphorylation sites and tau kinases, respectively.

Mitochondrial dysfunction and oxidative stress are both intimately associated with cell death in neurodegeneration. Mitochondrial oxidative stress in superoxide dismutase 2-deficient and APP expressing mice exacerbated amyloid burden and the hyperphosphorylation of tau at S396. Treatment with high doses of antioxidants prevented from tau hyperphosphorylation and neuropathology (Melov et al., 2007). Triple AD mice expressing mutant tau, APP and presenilin 1 developed tangles and Aβ plaques, and displayed deregulation of several mitochondrial proteins suggesting synergistic effects of Aβ and tau in perishing mitochondria (Rhein et al., 2009).

Undoubtedly, tau hyperphosphorylation is an important phenomenon in AD and other tauopathies and parallels the appearance of tau aggregates and NFTs, but despite great efforts, the underlying mechanisms that ultimately lead to toxicity and neurodegeneration remain elusive (Papanikolopoulou et al., 2010; Ambegaokar and Jackson, 2011). In recent years, it was hypothesized that the segregation of tau in intracellular aggregates is an escape route for the cell from excess amount of protein. Instead, tau oligomers were considered as toxic species that harm the cell and, ultimately, lead to cell death (Sahara and Avila, 2014).

Accumulation of phosphorylated (AT8, PHF-1, S422) tau oligomers was detected at human AD synapses concomitant with dysfunction of the UPS (Henkins et al., 2012; Tai et al., 2012). Use of a tau oligomer-specific antibody in human AD brain samples revealed that tau oligomers appear at early stages in AD, either before or after the manifestation of tau phosphorylation at specific epitopes (Lasagna-Reeves et al., 2012). Thus, aggregation of the hyperphosphorylated forms of tau into PHF structures could be neurotoxic by sequestering important cellular proteins, but it could also be neuroprotective by avoiding accumulation of toxic oligomeric tau.

### Physiological and pathological implications of tau phosphorylation

At normal levels of phosphorylation, tau contains 2–3 moles phosphate/mole of protein and is a soluble cytosolic protein (Khatoon et al., 1992). From the overall 85 phosphorylatable residues, approximately 30 residues are phosphorylated in normal tau proteins (Morishima-Kawashima et al., 1995a; Hanger et al., 2009a). Most of the tau phosphorylation sites are clustered in the proline-rich region, the microtubule binding repeats (MTBR) or MTBR-flanking domains.

Tau expression and phosphorylation are developmentally regulated. A single tau isoform is expressed in fetal human brain whereas six isoforms are expressed in adult human brain, with fetal tau corresponding to the shortest adult tau isoform. The degree of tau phosphorylation decreases during embryogenesis (Mawal-Dewan et al., 1994), which might be related to increasing neuronal plasticity in the early developmental process (Brion et al., 1993; Hanger et al., 2009a). PHF-tau contains 3–4 fold phosphates over the normal adult tau (Khatoon et al., 1992; Iqbal et al., 2013). In immature brain, as in PHFs, tau is phosphorylated at a large number of sites (Kenessey and Yen, 1993; Morishima-Kawashima et al., 1995b). However, as in adult brain, the phosphorylation in fetal tau is only partial. Phosphorylation of tau in PHFs is denominated as “hyperphosphorylation” which takes into account that other sites than the physiological ones are phosphorylated. This state is also referred to as “abnormal” or “pathological” phosphorylation.

MT dynamics are dependent on a balanced ratio between tau molecules and MT tracks. Either excess or poor binding of tau molecules, e.g., through dysregulation of the tau phosphorylation state, results in destabilization and breakdown of MT networks. This has a direct impact on MT function in the formation of the cytoskeletal architecture and as track for axonal and organelle transport, and is resumed in the “Tau-microtubule hypothesis” (Alonso et al., 1994).

Early studies clearly demonstrated that tau plays an important role in the establishment of neuronal polarity and axonal outgrowth (Caceres and Kosik, 1990). Neurite extension and retraction may be regulated by MARK and GSK-3β-mediated tau phosphorylation (Biernat et al., 2002; Sayas et al., 2002). It was speculated that phosphorylation of tau within the MTBR is necessary for appropriate neurite outgrowth whereas phosphorylation at SP and TP motifs within flanking domains retards neuronal differentiation (Biernat and Mandelkow, 1999). Tau, a cargo of kinesin, may displace other kinesin-based cargo indicating that the development and stabilization of axons are dependent on a balance of cytoskeletal elements (Dubey et al., 2008).

Overexpression of tau is known to compromise MT-dependent axonal transport in a phosphorylation-dependent manner (Sato-Harada et al., 1996). Co-expression of constitutively active GSK-3β exacerbated, whereas GSK-3β inhibition rescued vesicle aggregation and locomotor dysfunction in a *Drosophila* model (Mudher et al., 2004; Cowan et al., 2010b). Phosphorylation of tau at Y18 by the Fyn kinase was suggested to prevent the activation of the GSK-3β signaling cascade, thereby counteracting tau's inhibitory effect on anterograde fast axonal transport (Kanaan et al., 2012). These data suggest that the pathological over-activation of GSK-3β inhibits axonal transport through hyperphosphorylation of tau. In contrast, other studies showed that the inhibition of tau phosphorylation by GSK-3β inhibitors was associated with decreased mitochondrial transport and motility and increased mitochondrial clustering in cells (Tatebayashi et al., 2004; Llorens-Martin et al., 2011). Tau may control intracellular trafficking by affecting the frequencies of attachment and detachment of motors, in particular kinesin, to the MT tracks (Trinczek et al., 1999; Morfini et al., 2007). It was speculated that excess tau acts as transport block for vesicles and organelles which is reversed by removal of tau through MARK-mediated tau phosphorylation and subsequent detachment of tau from MTs (Thies and Mandelkow, 2007). However, detachment of tau from MT may also contribute to axonal transport blockage and neurodegeneration (Iijima-Ando et al., 2012). Dephosphorylation and phosphorylation cycles of tau, through the interplay of tau kinases and phosphatases, may serve as general mechanism to regulate tau's function to maintain a dynamic MT network for neurite outgrowth and axonal transport (Fuster-Matanzo et al., 2012; Mandelkow and Mandelkow, 2012). Interestingly, improper distribution of overexpressed tau in the somatodendritic compartment was shown to result in more numerous and densely packed MTs in axons and dendrites. Phosphomimic mutations of the AT8 epitope caused expansion of the space between MTs and may thereby contribute to axonal transport and mitochondrial movement defects (Thies and Mandelkow, 2007; Shahpasand et al., 2012). Furthermore, phosphorylated tau may sequester normal tau in neurites away from MTs leading to disruption of the microtubular cytoskeleton and demise of axonal transport (Niewiadomska et al., 2005; Cowan et al., 2010a; Iqbal et al., 2013).

Extracellular Aβ, shown to exacerbate the hyperphosphorylation of tau and NFT formation, was also suggested to modulate N-methyl-D-aspartate receptor (NMDAR) function and to induce excitotoxicity (Lauren et al., 2009). However, among the plethora of known Aβ-interacting molecules, the specific Aβ target and the intracellular propagation of the signal remain elusive. Prion protein was proposed as binding partner of Aβ but there is still controversy about the significance of this interaction (Balducci et al., 2010; Kessels et al., 2010; Chen et al., 2013a). Aβ induces the activation of Fyn which, in turn, increases the phosphorylation of a subunit of NMDARs dependent on the status of tau phosphorylation and tau localization at the post-synapse. After an initial increase, the number of surface NMDARs declined which resulted in dendritic spine loss and excitotoxicity (Um et al., 2012). The interaction of Fyn and tau, both forming a complex together with NMDAR, seems to modulate synaptic plasticity and to sensitize synapses to glutamate excitoxicity in AD (Ittner et al., 2010).

Phosphorylation of tau was also linked to altered turnover and proteolysis. The detection of ubiquitin immunoreactivity in tau inclusions was interpreted as failure of the ubiquitin proteasome system (UPS) to proteolytically degrade excess tau (Bancher et al., 1989). Proteasomal inhibition resulted in the accumulation of particularly hyperphosphorylated tau species (Shimura et al., 2004) and disruption of neuritic transport (Agholme et al., 2013). Inhibition of autophagy in neurons resulted in 3-fold accumulation of phosphomimic tau over wild type tau indicating that both, autophagic and proteasomal pathways, are responsible for the clearance of phosphorylated tau species (Rodriguez-Martin et al., 2013). Biochemical and morphological analysis of AD cortices revealed that tau becomes hyperphosphorylated and misfolded at presynaptic and postsynaptic terminals, in association with an increase in ubiquitinated substrates and proteasome components (Tai et al., 2012).

Many other mechanisms were suggested for the implication of tau hyperphosphorylation in tauopathies. Cell death was accompanied by expression of cell-cycle regulatory proteins in aged mice expressing human tau isoforms on a knockout background (Andorfer et al., 2003). Inappropriate re-entry to the cell cycle plays a role in AD and might be linked to hyperphosphorylation of tau via activation of cell-cycle relevant kinases (Delobel et al., 2002; Absalon et al., 2013). Abnormal interaction with the mitochondrial fission protein Drp1 might be causative for mitochondrial dysfunction and neuronal damage (Manczak and Reddy, 2012). DNA damage resulted in the activation of the checkpoint kinases Chk1 and 2, subsequent tau phosphorylation at AD-related sites, and enhancement of tau-induced neurodegeneration in human tau expressing *Drosophila* (Iijima-Ando et al., 2010). Immunohistochemical analysis of AD brains revealed that tau is truncated at D421, and that this cleavage occurs after conformational changes detected by the Alz-50 antibody but precedes cleavage at E391 (Guillozet-Bongaarts et al., 2005). Accumulation of D421 and E391-truncated species occurs early in the disease and correlates with the progression in AD (Basurto-Islas et al., 2008). In transgenic mice, truncation of tau was shown to drive pre-tangle pathology (McMillan et al., 2011). In cells, hyperphosphorylation of tau at several residues and cleavage of tau at D421, the preferential cleavage site of caspase-3, enhanced the secretion of tau. This was suggested as potential mechanisms for the propagation of tau pathology in the brain and tau accumulation in the CSF (Plouffe et al., 2012).

### Kinases involved in tau phosphorylation

**Figure 3:**
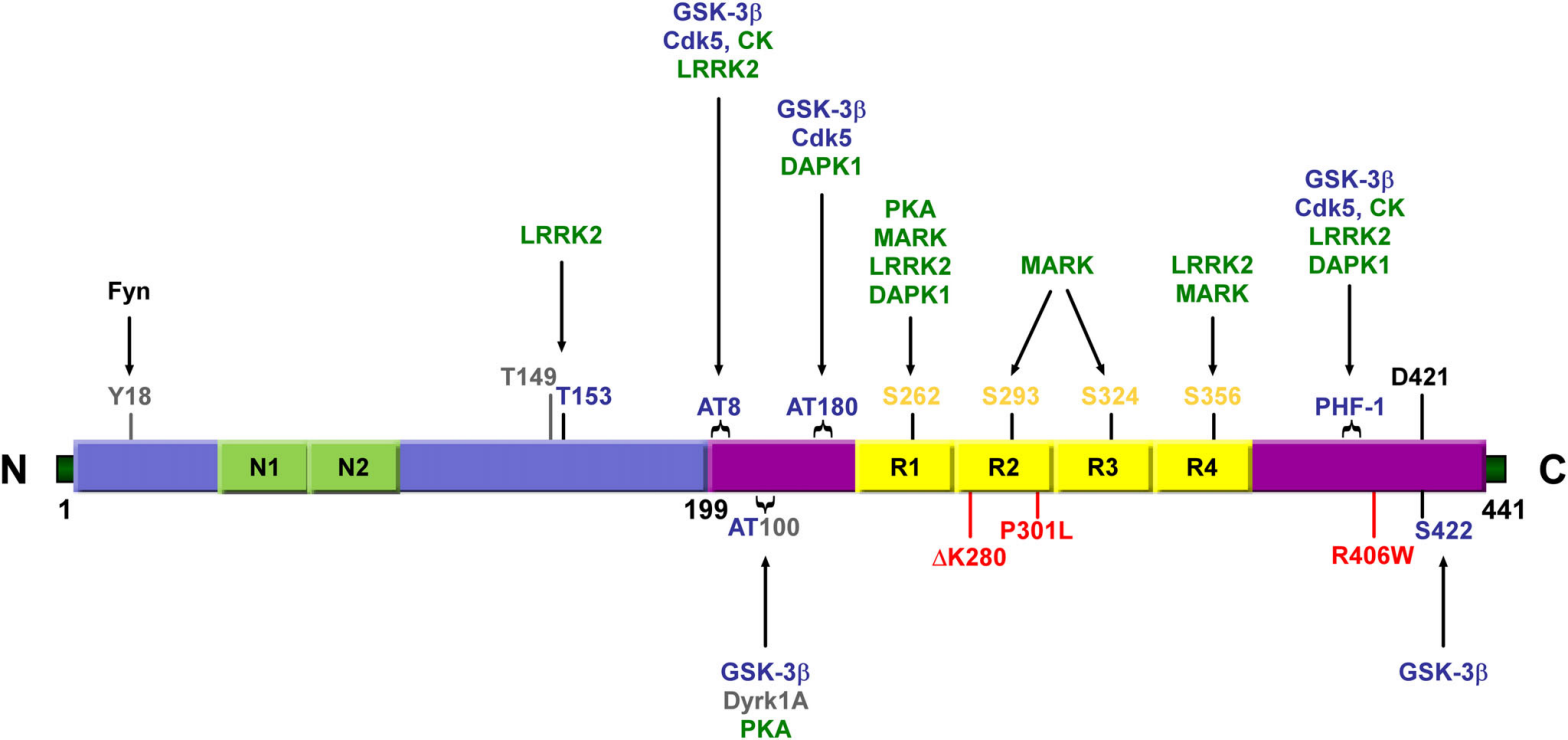
**Schematic representation illustrating the various residues in the longest isoform of tau that can be phosphorylated**. SP/TP motifs (represented in blue), KXGS motifs (represented in yellow), and other sites (represented in gray) can be phosphorylated by proline-directed kinases (represented in blue) and non-proline directed Ser/Thr kinases (represented in green). Antibody epitopes AT8, AT100, AT180, and PHF-1 comprise dual and triple serine/threonine residues (indicated by brackets). Some mutations associated with FTDP-17 are shown in red. Alternative splicing of N1, N2, and R2 generates the six different isoforms of tau. N1, N2, N-terminal inserts 1 and 2; R1-R4,MT binding repeats 1–4; GSK-3β, Glycogen synthase kinase 3β; Cdk5, Cyclin-dependent kinase 5; CK, casein kinase; MARK, microtubule affinity-regulating kinase; LRRK2, leucine-rich repeat kinase 2; DAPK, Death-associated protein kinase; Dyrk1A, dual-specificity protein kinase.

Similar to the pattern of tau hyperphosphorylation, the idea of a distinct signature-specific pattern of tau kinase activation emerged (Duka et al., 2013). Several attempts were done to identify the responsible kinases and the corresponding phosphorylation sites of tau (Table 2). However, most of the kinases phosphorylate several residues of tau, and most tau phosphorylation sites are targets of more than one kinase (Figure 3). In addition, the existence of priming, meaning that the phosphorylation at one site facilitates phosphorylation at another site, and feedback events to regulate the overall level of tau phosphorylation, hamper the assignment of a specific phosphorylation site to a particular (dys-)function of tau (Bertrand et al., 2010; Kiris et al., 2011).

Numerous kinases, including more than 20 serine/threonine kinases, were shown to phosphorylate tau *in vitro* but their relevance in AD is still under investigation (Hanger et al., 2009b; Cavallini et al., 2013).

The proline-directed kinase GSK-3β was particularly associated with the formation of PHFs and NFTs and proposed as key mediator in the pathogenesis of AD (Hooper et al., 2008; Terwel et al., 2008; Ma, 2014; Medina and Avila, 2014). GSK-3β targets tau at SP/TP sites, including the epitopes PHF-1, AT8, AT180, AT100, S404 and S413 (Pei et al., 1999; Medina and Avila, 2014). Alterations in GSK-3β levels were associated with changes in tau phosphorylation in several cell and animal models (Hernandez et al., 2013). Stress stimuli such as mitochondrial toxins or oxidative stress to mimic conditions in neurodegenerative disorders resulted in increased GSK-3β-mediated phosphorylation of tau, reduced cell metabolic activity and MT destabilization (Hongo et al., 2012; Selvatici et al., 2013). Other studies position GSK-3β as prominent player in the pathogenesis of AD beyond its role as tau phosphorylating kinase. Tau-P301Lx GSK-3β mice developed severe forebrain tauopathy with tangles in the majority of neurons but in the absence of tau hyperphosphorylation (Muyllaert et al., 2006). In a *Drosophila* model, co-expression of a GSK-3β homolog and human tau led to increased toxicity more likely due to the fact that GSK-3β is a pro-apoptotic protein than due to increased tau phosphorylation (Jackson et al., 2002).

The serine/threonine kinase Cdk5 plays important roles in neuronal development and migration, neurite outgrowth, and synaptic transmission, and is implicated in the pathogenesis of AD (Cheung and Ip, 2012; Shukla et al., 2012). Immunoreactivity of Cdk5 in several brain regions in AD was associated with pre-tangle and early NFT stages, and colocalized with AT8-positive tau in a subset of neurons (Pei et al., 1998; Augustinack et al., 2002). Cdk5 activity was found to be higher in AD than control cases probably due to the conversion of the Cdk5 activator p35 into the constitutive active form p25 (Lee et al., 1999; Patrick et al., 1999; Shukla et al., 2012).

Mice overexpressing human p25/Cdk5 displayed enhanced Cdk5 activity, hyperphosphorylation of tau, and cytoskeletal disorganization (Ahlijanian et al., 2000). The activation of Cdk5 along with overexpression of mutant tau was associated with tau hyperphosphorylation and tangle formation (Noble et al., 2003). APPswe mice showed increased Cdk5 activity due to increases in p25 levels, and substantial phosphorylation of tau at AT8 and PHF-1 epitopes linking Aβ pathology to tau hyperphosphorylation via increased Cdk5 activity (Otth et al., 2002). Furthermore, Cdk5 was suggested to be linked to GSK-3β. Mice expressing human p25 showed elevated Aβ levels but decreased phospho-tau levels and reduced GSK-3β activity. Administration of Cdk5 inhibitors reduced Aβ production but did not alter the phosphorylation of tau suggesting that Cdk5 predominantly regulates APP processing, whereas GSK-3β plays a dominant role in tau phosphorylation (Wen et al., 2008; Engmann and Giese, 2009). The crosstalk between Cdk5 and mitogen-activated protein kinase (MAPK) pathways suggests a connection with neuronal apoptosis and survival signaling (Sharma et al., 2002; Zheng et al., 2007). Dysregulation of the MAPK signaling pathways, comprising the three signaling cascades extracellular signal regulated kinase (ERK), p38, and c-Jun N-terminal kinase (JNK), was suggested to be implicated in AD and other neurodegenerative disorders (Kim and Choi, 2010). In the course of AD, ERK and JNK are activated throughout all stages, and p38 in mild to severe cases (Braak stages III to VI) (Pei et al., 2001; Zhu et al., 2001). p38 and JNK immunoreactivity were associated with neurons containing neuritic plaques, neuropil threads, and NFTs, structures that were also recognized by antibodies raised against phosphorylated PHF-tau (Hensley et al., 1999; Atzori et al., 2001).

The kinases MARK1-4 are non-proline directed kinases that are involved in the establishment of neuronal polarity and the regulation of neurite outgrowth (Biernat et al., 2002; Matenia and Mandelkow, 2009; Reiner and Sapir, 2014). MARKs are named after their ability to regulate the affinity of tau to MTs through phosphorylation (Drewes et al., 1997). Importantly, MARKs phosphorylate tau within the KXGS motifs, particularly at S262, which phosphorylation is detected early in the course of AD. Expression of MARK2 and MARK4, as well as the interactions of these kinases with tau, were significantly enhanced in AD brains, correlated with the Braak stages of the disease, and were associated with NFTs (Chin et al., 2000; Gu et al., 2013a).

In transgenic *Drosophila*, overexpression of the *Drosophila* homolog Par-1 was associated with increased phosphorylation and enhanced toxicity of human tau. Loss of Par-1 function and mutation of tau at the Par-1 directed phosphorylation sites (S262, S356) rescued from tau-induced toxicity. Interestingly, Par-1 phosphorylation of tau was a prerequisite for downstream phosphorylation through GSK-3β and Cdk5, and the generation of disease-associated phosphorylation epitopes (Nishimura et al., 2004). Activation of MARK2 rescued from synaptic decay caused by overexpression and improper distribution of tau in the somatodendritic compartment (Mandelkow et al., 2004; Thies and Mandelkow, 2007). However, overexpression of MARK4 resulted in tau hyperphosphorylation and loss of spines, which also manifested after Aβ treatment. Therefore, MARKs may have regulatory functions in spine morphology and synaptic transmission, but may also act as critical mediators in Aβ-induced toxicity on synapses and dendritic spines (Zempel et al., 2010; Hayashi et al., 2011; Yu et al., 2012). Furthermore, the phosphorylation of tau by MARK was suggested to inhibit tau's assembly into PHFs (Schneider et al., 1999), contradictory to the hypothesis that the pool of hyperphosphorylated, MT-unbound tau assembles into PHFs. Phosphorylation of tau at SP/TP sites has low impact on the tau-MT binding and is observed in AD, dissociating the detachment of tau from MTs from the likability to assemble into PHFs. GSK-3β was shown to phosphorylate MARK2 at two different sites, the activatory T208 and the inhibitory S212, thereby modulating the phosphorylation of tau, particularly at S262 (Kosuga et al., 2005; Timm et al., 2008). MARK1/2 activity was also regulated by the death domain of DAPK. DAPK activated MARK and promoted the phosphorylation of tau but also seems to act via MARK-independent pathways on T231, S262, and S396 of tau (Wang et al., 2010; Duan et al., 2013). Moreover, DAPK induced rough eye and loss of photoreceptor neurons in a *Drosophila* model, in part through the activation of the *Drosophila* ortholog Par-1 (Wu et al., 2011b). PKC was described as negative regulator of MARK2, playing an important role in neuronal polarity (Chen et al., 2006).

CK1 and CK2 are serine/threonine-selective protein kinases. Overall CK2 immunoreactivity is reduced in the brain of AD cases although NFTs stain very strong with anti-CK2 antibodies (Iimoto et al., 1990). CK-1δ is upregulated in AD brain, correlating with the degree of regional pathology. CK-1δ colocalizes with NFTs, neuropil threads and dystrophic neurites (Yasojima et al., 2000). In cells, CK1δ inhibition reduced the phosphorylation of tau at S396/S404 by more than 70%. Exogenous expression of CK1δ increased tau phosphorylation at S202/T205 and S396/S404 and reduced tau-MT binding (Lee and Leugers, 2012).

Several sites in PHF-tau are targeted by CK1 in concert with other kinases such as GSK-3β and protein kinase A (PKA). Moreover, three sites, S113, S238, and S433, were phosphorylated only by the action of CK1δ suggesting a relevant role of this kinase in tau pathology (Hanger et al., 2007). Synthetic Aβ was reported to stimulate the activities of CK1 and CK2 and to mediate phosphorylation of the substrate casein *in vitro* (Chauhan et al., 1993). Aβ production was increased in cells with exogenous expression of constitutively active CK1 and reduced by CK1 specific inhibition (Flajolet et al., 2007).

Dyrk1A is upregulated in AD, Down's syndrome, and Pick's disease. Dyrk1A immunoreactivity was observed in the cytoplasm and nucleus of scattered neurons, and detected in sarkosyl-insoluble PHF fractions. Overexpression of Dyrk1A in transgenic mice led to increased tau levels in the brain and accumulation in NFTs (Wegiel et al., 2008). Direct phosphorylation of tau at T212 by Dyrk1A still lacks evidence *in vivo*.

LRRK2, a putative kinase, gained interest in recent years due to its genetic association with both, inherited and sporadic PD, and a possible overlap to AD (Zhao et al., 2011; Ujiie et al., 2012). The first hints for an involvement of LRRK2 in tau pathology were given in transgenic mice expressing mutant LRRK2. These animals showed increased tau phosphorylation at T149, T153, and the AT8, CP13, 12E8 and PHF-1 epitopes, tau mislocalization in cell bodies and the neuropil, and tau aggregation (Li et al., 2009, 2010; Melrose et al., 2010; Bailey et al., 2013). Correspondingly, phosphorylation of tau at the AT8 epitope was decreased in LRRK2 knock-out mice (Gillardon, 2009). Several studies imply that LRRK2 phosphorylates and activates other kinases and signal transduction pathways, thereby contributing to enhanced tau phosphorylation, mislocalization, and dendritic degeneration (Gloeckner et al., 2009; Lin et al., 2010; Chen et al., 2012).

Several other kinases may be implicated in the pathology of AD, anticipated from their aberrant activity in human brain (Martin et al., 2013). In general, dysregulation of kinases is likely to be responsible for the hyperphosphorylation of tau, abnormal tau-MT binding, tau mislocalization, and tau assembly into PHFs. However, it is still not known, which phosphorylation sites of tau are the most critical ones, which kinases are the main players, and how these processes are mechanistically linked to toxicity in AD and other neurodegenerative disorders.

## Phosphorylation as a target for therapeutic intervention

Our current lack of understanding of the precise molecular mechanisms underlying neurodegenerative disorders has limited our ability to develop effective therapeutic strategies. Targeting phosphorylation of aSyn and tau can impact on several mechanisms associated with the pathogenesis of AD, PD and other neurodegenerative disorders. Thus, reducing aberrant protein phosphorylation and protein levels, preventing protein aggregation, eliminating amyloidogenic species and preventing spreading of pathology are all potentially beneficial and will be discussed below.

### Kinase inhibition

Although the precise consequences of aSyn phosphorylation remain to be fully understood, it is evident that pS129 correlates with disease progression, is present in the pathological hallmark lesions of synucleinopathies and can have detrimental functional consequences. Thus, inhibition of the relevant kinases might constitute a possible therapeutic strategy. Inhibitors of PLKs have been developed for oncology indications and tested *in vivo*. A PD mouse model of subject to a treatment with the specific PLK inhibitor BI2356 presented reduced pS129 aSyn (Inglis et al., 2009). However, the long-term safety of such strategy is currently unclear, as the kinases that phosphorylate aSyn have ubiquitous distribution and because there is evident redundancy in the types of kinases phosphorylating same residues in aSyn. In the particular case of PLK2, recent data suggest that enhancing the kinase activity, instead of inhibiting, might also prove worth investigating further, as PLK2 suppresses aSyn toxicity *in vivo* by promoting autophagy-mediated degradation of pS129 aSyn (Oueslati et al., 2013).

The pharmacological inhibition of c-Abl is also emerging as an attractive therapeutic strategy, as it was found to be neuroprotective in animal models of PD (Ko et al., 2010; Imam et al., 2011, 2013; Hebron et al., 2013a; Mahul-Mellier et al., 2014), by promoting aSyn degradation (Hebron et al., 2013a,b; Mahul-Mellier et al., 2014). Interestingly, c-Abl inhibition also targets hyperphosphorylated tau for degradation (Hebron et al., 2013a) and inhibits β-amyloid production in rat neuronal primary cultures and in guinea pig brains (Netzer et al., 2003). Therefore, this kinase is a promising target for the treatment of both, PD and AD.

Inhibition of GSK-3β activity by chemical compounds, antisense RNAs and kinase-dead mutations or reduction of GSK-3β levels were the most promising attempts to decrease the phosphorylation of tau at critical residues. This was shown to counteract neuronal death, reduce oxidative stress, and improve learning and memory (Hernandez et al., 2013; Medina and Avila, 2014). GSK-3β was suggested as missing link between Aβ and tau pathology. Small molecule inhibitors of GSK-3β might be potent to reduce Aβ-induced tau hyperphosphorylation (Noh et al., 2013; Ye et al., 2013). Given the contribution of other kinases to tau hyperphosphorylation, effective treatment may require multiple kinase targeting (Mazanetz and Fischer, 2007; Tell and Hilgeroth, 2013; Pinsetta et al., 2014).

### Immunological targeting of phosphorylated proteins

The consequences of loss of aSyn function in PD are not completely clarified. Some studies suggest that a substantial reduction in the levels of aSyn could have potential harmful effects in the CNS. In fact, aSyn knockout mice show some loss of dopaminergic nigrostriatal terminals with aging (Al-Wandi et al., 2010). Moreover, aSyn seems to play an important role holding together the SNARE complex, suggesting that excessive reduction of this abundant protein in the nervous system could lead to deleterious effects. Nevertheless, therapeutic strategies involving immunization or promoting clearance of aSyn excess are attractive and have been recently considered. Studies exploring immunization as a potential therapeutic were performed in mice models of PD and achieved promising results (Masliah et al., 2005, 2011). However, to our knowledge, the use of phospho-specific antibodies against aSyn was not explored so far.

Targeting specific phospho-tau sites through passive immunization may be useful to slow, or even reverse, the progression of a disease. Antibody uptake resulted in reduced tau phosphorylation, and clearance of pathological tau protein in brain slices up to significantly improved cognitive performance in Thy-Tau22 transgenic mice (Troquier et al., 2012; Gu et al., 2013b). However, repeated immunization of mice with phospho-tau peptides may cause neuroinflammation (Rozenstein-Tsalkovich et al., 2013).

### Activation of phosphatases

Another possible therapeutic strategy could involve restoring or increasing the activity of specific phosphatases. Although phosphatases are thought to be less appealing drug targets, since they are considered less specific than kinases, increasing evidence suggests that they might be “druggable” proteins. On the other hand, the lower level of redundancy may be seen as an advantage. In fact, pharmacological induction of the phosphoprotein phosphatase 2A (PP2A) by eicosanoyl-5-hydroxytryptamide resulted in dephosphorylation of aSyn at S129, inhibition of aSyn aggregation with concomitant improved neuronal integrity, reduction in inflammation and amelioration of behavioral deficits in an aSyn transgenic mouse model, (Lee et al., 2011).

Dysregulated phosphatase activity also seems to be partially responsible for tau pathology. A number of pharmacological agents, such as the FDA-approved drug memantine, prevented okadaic acid or calyculin-induced PP2A inhibition and tau phosphorylation (Li et al., 2004; De Los Rios et al., 2010; Kickstein et al., 2010; Yang et al., 2011).

### Modulation of protein clearance via phosphorylation

The induction of aSyn degradation through clearance pathways is also seen as an attractive therapeutic strategy. aSyn can be degraded by direct proteolysis, by the UPS, by chaperone-mediated autophagy, or by general autophagy (Lashuel et al., 2013). As discussed earlier, phosphorylation of Y39 by c-Abl impairs aSyn degradation by autophagy and proteasome (Mahul-Mellier et al., 2014), while PLK2 phosphorylation at S129 promotes selective autophagic aSyn clearance (Oueslati et al., 2013). Likewise, inhibiting c-Abl and enhancing PLK2 activity are two promising therapeutic approaches.

Several studies indicate that the reduction of tau phosphorylation was paralleled by an overall decrease in protein levels. Moreover, targeting unphosphorylated tau protein had deleterious effects since tau is a normal component of the cytoskeleton (Rosenmann et al., 2006). Additionally, reduced protein levels were achieved through interference with the UPS and the lysosomal / autophagic pathways. Immunization using phospho-specific antibodies against tau epitopes resulted in reduced levels of tau protein and clearance of tau aggregates (Asuni et al., 2007; Boimel et al., 2010; Boutajangout et al., 2010, 2011). Interestingly, memantine that inhibited the phosphorylation of tau at some epitopes in hippocampal slices also reduced tau aggregation (Li et al., 2004). The small molecule IU1, a potent and selective inhibitor of the deubiquitinating enzyme ubiquitin specific peptidase 14 (USP14), enhanced the degradation of tau (Lee et al., 2010). The underlying mechanisms are not clear because USP14-deficient mice showed no alterations in tau degradation and actually increased amounts of phosphorylated tau (Jin et al., 2012). Chronic treatment with lithium chloride, a direct inhibitor of GSK-3β, reduced tau pathology by promoting ubiquitination (Nakashima et al., 2005).

Positive lysosomal modulation was described for several factors and may be an attempt of cells to clear amyloidogenic species such as tau and aSyn oligomers (Lee et al., 2004b; Butler et al., 2006; Bahr et al., 2012). Methylene blue (MB) was shown to induce autophagy and to reduce total and phosphorylated levels of tau. Although MB administration improved cognitive performance in tau transgenic mice, a reversal of already existing NFTs was disputed (Congdon et al., 2012; Spires-Jones et al., 2014). Interestingly, MB was the first identified direct tau aggregation inhibitor (Duff et al., 2010). Modified versions of this substance with greater tolerability and better absorption are underway in clinical trials (Wischik et al., 2013). Lithium chloride prevented tau aggregation in cultured neurons probably through decreased tau protein levels as outlined above (Rametti et al., 2008). In recent studies, pre-filamentous aSyn and tau oligomers rather than aSyn fibrils and NFTs were considered as toxic species questioning the usefulness of aggregation inhibitors (Castillo-Carranza et al., 2013; Crowe et al., 2013; Lesne, 2013). Furthermore, at higher doses, some of the *in vitro* tested substances showed severe side effects on the normal biology of tau and its MT stabilization in cell culture and brain slices (Duff et al., 2010).

### Modulation of pathology spreading via phosphorylation

Recent studies strongly suggest that disease progression in AD, PD, and other neurodegenerative disorders may, at least in part, be due to cell-to-cell transmission of amyloidogenic species. Thus, the elimination of these toxic species may help to slow or contain neurodegeneration.

The mechanisms behind the spreading of aSyn are not fully understood. However, aSyn secreted by neurons could strongly contribute to cell-to-cell propagation (Marques and Outeiro, 2012; Eisbach and Outeiro, 2013). The relationship between the phosphorylated status of aSyn and its secretion are currently unknown and require investigation, but if correlation exists then phosphorylation might be targeted to prevent the spreading of aSyn pathology.

Immunization with cell-penetrating phospho-tau antibodies was already discussed as a therapeutic approach to clear from toxic tau species. Similarly, antibodies that remain in the extracellular space may inhibit spreading and this includes both, anti-tau and anti-Aβ antibodies (Giacobini and Gold, 2013; Liu et al., 2014). Lowering the intracellular amyloid burden through RNA interference or by drugs may entail reduced secretion of Aβ and thereby lessen tau pathology (Chen et al., 2013b; Spilman et al., 2013). However, some of these treatments failed in clinical trials (Doody et al., 2014). Importantly, extracellular soluble tau was also shown to initiate spread of tau pathology and may be a plausible target for treatment (Michel et al., 2014).

## Conclusions and future perspectives

While it is clear that phosphorylation of aSyn and tau is relevant in the context of their aggregation and toxicity, there is still no final consensus on the precise contribution this type of PTM has toward the disease process. For example, there is still no consensus on whether aSyn phosphorylation is a cause or a consequence of aggregation, or whether phosphorylation is neurotoxic or neuroprotective. Furthermore, only a few phosphorylation sites have been confirmed in human tissue so far, so there may be other sites relevant to human pathology that remain to be identified. Tau phosphorylation regulates tau's function in many ways while abnormal phosphorylation of tau is neurotoxic. The relationship between tau phosphorylation and aggregation is clearly complex with evidence that tau phosphorylation precedes, prevents or is irrelevant to its aggregation. It will also be important to explore the cross-talk between different PTMs, as this will likely have a strong impact on our understanding of the biology/pathobiology of different proteins associated with neurodegeneration. In addition, the identification of the kinases and phosphatases involved in the phosphorylation/dephosphorylation of aSyn and tau will certainly open novel possibilities for pharmacological intervention. Ultimately, solving the problems and inconsistencies surrounding the phosphorylation of these important players in AD and PD will be essential for advancing the development of novel therapeutic strategies.

### Conflict of interest statement

The authors declare that the research was conducted in the absence of any commercial or financial relationships that could be construed as a potential conflict of interest.
